# Scoring model based on the signature of non-m6A-related neoantigen-coding lncRNAs assists in immune microenvironment analysis and TCR-neoantigen pair selection in gliomas

**DOI:** 10.1186/s12967-022-03713-z

**Published:** 2022-10-29

**Authors:** Wenbo Zhao, Yibo Wu, Feihu Zhao, Zhiyi Xue, Wenyu Liu, Zenxin Cao, Zhimin Zhao, Bin Huang, Mingzhi Han, Xingang Li

**Affiliations:** 1 Department of Neurosurgery, Qilu Hospital, Cheeloo College of Medicine and Institute of Brain and Brain-Inspired Science, Shandong University, Jinan, 250012 China; 2 Jinan Microecological Biomedicine Shandong Laboratory and Shandong Key Laboratory of Brain Function Remodeling, Jinan, 250117 China; 3 Medical Integration and Practice Center, Cheeloo College of Medicine, Shandong University, Jinan, 250012 China

**Keywords:** Glioma, LncRNA, Non-m6A modification, Machine learning, Neoantigen, Immunotherapy

## Abstract

**Background:**

Small peptides encoded by long non-coding RNAs (lncRNAs) have attracted attention for their various functions. Recent studies indicate that these small peptides participate in immune responses and antigen presentation. However, the significance of RNA modifications remains unclear.

**Methods:**

Thirteen non-m6A-related neoantigen-coding lncRNAs were selected for analysis from the TransLnc database. Next, a neoantigen activation score (NAS) model was established based on the characteristics of the lncRNAs. Machine learning was employed to expand the model to two additional RNA-seq and two single-cell sequencing datasets for further validation. The DLpTCR algorithm was used to predict T cell receptor (TCR)-peptide binding probability.

**Results:**

The non-m6A-related NAS model predicted patients’ overall survival outcomes more precisely than the m6A-related NAS model. Furthermore, the non-m6A-related NAS was positively correlated with tumor cells’ evolutionary level, immune infiltration, and antigen presentation. However, high NAS gliomas also showed more PD-L1 expression and high mutation frequencies of T-cell positive regulators. Interestingly, results of intercellular communication analysis suggest that T cell-high neoplastic cell interaction is weaker in both of the NAS groups which might arise from decreased IFNGR1 expression. Moreover, we identified unique TCR-peptide pairs present in all glioma samples based on peptides encoded by the 13 selected lncRNAs. And increased levels of neoantigen-active TCR patterns were found in high NAS gliomas.

**Conclusions:**

Our work suggests that non-m6A-related neoantigen-coding lncRNAs play an essential role in glioma progression and that screened TCR clonotypes might provide potential avenues for chimeric antigen receptor T cell (CAR-T) therapy for gliomas.

**Supplementary Information:**

The online version contains supplementary material available at 10.1186/s12967-022-03713-z.

## Background

Gliomas are the most prevalent brain tumor with dismal outcomes, with a median survival time of 12–15 months after diagnosis and a 5-year survival rate of no more than 3% [1, 2]. One factor contributing to gliomas’ refractoriness is relatively low immune responses, namely responses that are “immunologically cold” or immunosuppressive. Although some of immunotherapy agents like depatuxizumab [3] manifested curing effects in recurrent glioblastoma patients, other agents including bevacizumab [4, 5] and PD-1/PD-L1 antibodies such as nivolumab [6, 7] and the like, which have been proven effective for the treatment of other tumors, are of limited value for primary glioma patients. Besides, although chimeric antigen receptor T cell (CAR-T) therapy was approved for the treatment of B-cell malignancies by the American Food and Drug Administration, it does not have a satisfying effect on solid tumors such as gliomas, which is partly due to the immunosuppressive microenvironment inside solid tumors [8]. However, the personalized tumor vaccine that is based on neoantigens produced by gliomas provides a new avenue for glioma treatment [9]. However, identification of the neoantigens appropriate for use in CAR-T therapy for gliomas is challenging.

In most situations, cancers arise from an accumulation of genetic mutations or damage to DNA [10]. Neoantigens derive from these non-synonymous genetic mutations, including single nucleotide variations (SNV), chromosomal deletions and insertions, gene fusion, and alternative splicing [11]. As the presented neoantigens might elicit T-cell-mediated anti-tumor immunity specifically against tumor cells, they are considered promising immunotherapy targets [12]. Recently, neoantigens derived from non-coding regions of RNAs have received attention [13, 14]. Of these, long-non-coding RNAs (lncRNAs) are especially notable for their crucial role in modulating immune responses [15, 16] and their ability to translate short peptides [17]. LncRNAs have also been reported to translate neoantigens that can be presented by major histocompatibility complex class 1 molecules (MHC I), which contributes to essential cellular immunosurveillance [17] and expands the range of immunopeptidomes that can be targeted for immunotherapy [18].

The translation of lncRNAs can be modulated by many factors, including small or short open-reading frames (smORF or sORF) [19], eIF4E [20], and N6-methyladenosine (m6A) modification. lncRNAs lack canonical ORF codes for over 100 amino acids [19]. Therefore, they are considered untranslatable until sORF or smORF, which encode small peptides, are identified inside them [21]. eIF4E is a translation initiation factor that weakly binds to 5′ caps of RNA after phosphorylation, which induces inhibition of mRNA translation and facilitates interaction between lncRNA and ribosomes [22]. Additionally, m6A modification has been proven to affect mRNA translation [23]. Studies have shown that m6A-modified sites serve as translation initiation sites for circular RNA [21]. Recent studies have provided further evidence that lncRNA translation that produces micropeptides is also affected by m6A modification [24]. Moreover, TransLnc [25] and LncPep [26], two recently created databases, have recorded the potentially translatable lncRNAs that have been identified through experimental evidence or algorithmic deduction. Both databases use m6A modification as an index in the evaluation of lncRNA translation potential. By calculating micropeptides' binding affinities to MHC I and MHC II, TransLnc also evaluated their potential to be presented by MHC complex so that they might act as neoantigens. Results indicate that m6A plays an important role in both lncRNA translation and neoantigen production from lncRNA. Moreover, select RNA modification processes, such as pseudouridine (Ψ), N1-methyladenosine (m1A), and 5-methylcytosine (m5C), also participate in the modulation of mRNA translation, the launching of immune responses [27, 28], and the modification of lncRNA [29–32]. However, whether these non-m6A modifications are related to lncRNA-mediated immune processes and neoantigen production has yet to be explored.

Here, we investigate the signatures of neoantigen-coding lncRNAs that could be related to m6A or non-m6A modifications in the TransLnc database. We compared two sets of lncRNAs by analyzing gene expression data of gliomas from The Cancer Genome Atlas (TCGA) and discovered that the signature of non-m6A-related neoantigen-coding lncRNAs has better efficacy in the prognostic model. We then established a scoring model using a clustering model of the non-m6A-related neoantigen-coding lncRNAs’ signature. This score is highly correlated with immune infiltrations, glioma cell development, glioma patient prognoses, and tumor mutation burden (TMB) of T-cell positive regulators. After investigating the correlation between this score and gliomas’ T cell receptor (TCR) repertoires, we found that the score model is positively correlated with the expression levels of widely expressed neoantigen-active TCR clonotypes. In summary, these results indicate that the model of non-m6A-related neoantigen-coding lncRNAs is a promising tool for determining glioma patient prognoses, and it also provides widely targetable T cell clonotypes for potential CAR-T therapy for the treatment of gliomas.

## Methods

### Data preparation

mRNA sequencing data were downloaded from TCGA database (https://www.cancer.gov/about-nci/organization/ccg/research/structural-genomics/tcga), and validation data were downloaded from the Chinese Glioma Genome Atlas (CGGA) database (http://www.cgga.org.cn/). Two datasets from the CGGA, “mRNAseq_325” and “mRNAseq_693”, were employed; these are labelled herein as CCGA325 and CCGA693, respectively. There were 169 glioblastoma multiforme (GBM) samples and 529 low-grade glioma (LGG) samples in the training cohort of TCGA dataset, and there were 139 GBM samples and 186 LGG samples in the CGGA325 dataset. Also, 249 GBM samples and 444 LGG samples were included in the CGGA693 dataset. Verhaak classification was performed, as described above [33].

Single-cell sequencing (scSeq) data were obtained from the Gene Expression Omnibus (GEO) database (https://www.ncbi.nlm.nih.gov/geo/), from which GSE84465 was selected for analysis. This dataset contained 3589 cells. Furthermore, another dataset from the CGGA database containing 6148 cells was also included. GSE129671 was used for SCENIC analysis. The “Seurat” package from R was applied to normalize the count data from these datasets, and the “FindMarkers” function was used to identify unique gene markers in every cluster. Cell distribution was displayed using the “tSNE" or “umap” functions. The cells were annotated as described in a study that also used the GSE84465 dataset [34] after undergoing slight modifications.

TCR sequencing data were acquired from the GEO database, and two datasets, GSE79338 and GSE188620, were used for analysis. The GSE79338 dataset contained TCR data from normal brain tissue as LGG and GBM were used to explore unique TCR patterns or clonotypes in gliomas as compared to normal brain tissue in this study. Two samples, GBM09 and GBM13, were excluded as they had relatively fewer TCR clonotypes than other GBM samples. The GSE188620 dataset contained TCR sequencing data before and after GBM-cell lysate vaccination, and it was used in this study for the validation of selected TCR patterns.

### Neoantigen activation score (NAS) model

In accordance with the literature, the high- to intermediate-level modifications (Ψ and m5C) and ultra-low-level modifications (m1A) were used as the main non-m6A modifications in this study [35]. Their regulators were identified in previous studies [36–38]. The cluster model was constructed using the “ConsensusClusterPlus” package in R. We used “km” (k-means) as the cluster algorithm and “euclidean” as the distance function. Two clusters, scored with continuous numbers between 2 and 9, were considered to have the best clustering results that showed the highest clustering reliability (Additional file 2: Fig. S2A).

Next, the differential expression genes (DEGs) were identified by comparing cluster 1 to cluster 2 using the “limma”, “edgeR” and “DESeq2” packages in R with a threshold of false discovery rate (FDR) < 0.05 and |log2(fold change)|> 1 (Additional file 12: Table S1 and Additional file 13: Table S2). For DEGs of CGGA693 in non-m6A-related NAS model, no gene could meet this threshold in limma and DESeq2 analyses so we used FDR < 0.05 and |log2(fold change)|> 0.4 in edgeR to keep the number of DEGs similar to CGGA325 dataset. Then the DEGs were confirmed as the intersection of these three parts. A univariate cox regression was then conducted in order to identify the genes associated with significant survival outcomes. Following this, the principal components of these significant genes were calculated, and TCGA samples’ NASs were calculated using the following formula according to a previous study [34].

$$\mathbf{N}\mathbf{A}\mathbf{S}=\mathbf{k}\mathbf{*}\sum \left({\mathbf{G}\mathbf{e}\mathbf{n}\mathbf{e}}_{\mathbf{H}\mathbf{R}>1}\mathbf{*}\left(\mathbf{P}\mathbf{C}1+\mathbf{P}\mathbf{C}2\right)\right)-\mathbf{k}\mathbf{*}\sum \left({\mathbf{G}\mathbf{e}\mathbf{n}\mathbf{e}}_{\mathbf{H}\mathbf{R}<1}\mathbf{*}(\mathbf{P}\mathbf{C}1+\mathbf{P}\mathbf{C}2)\right)$$

Gene_HR>1_ and Gene_HR<1_ represent the expression levels of genes with a hazard ratio (HR) that is higher or lower than 1 in survival analyses, respectively. Gene expression levels are in fragments per kilobase of transcript per million mapped reads (FPKM). To minimize the NAS value without altering its prognostic efficacy, k is set as 0.0001 to keep most of the NAS value ranging from ten to thousands.

After this, the characteristics of cluster 1 and 2 were identified through the use of the support vector machine (SVM) that is embedded in R package “e1071”, indicated by the function “svm”. For parameters of this function, the kernel “radial” was applied, and k-fold cross was set to 10. The randomly selected 520 samples (75% of all) in TCGA data were used as training phase data while the rest 174 samples (25% of all) were used as testing phase data. There were two clusters resulting from SVM-based prediction: cluster 1 and cluster 2. The cluster model was then reproduced in the CGGA325 and CGGA693 datasets using the “predict” function. The NASs were calculated through a similar process.

### Overall survival outcome prediction

The TCGA, CGGA325, and CGGA693 samples were divided into cluster 1 and cluster 2 and into high and low NAS groups by the cluster model or by the NAS. Kaplan-Meier analysis was used to determine the different groups' overall survival time. The receiver operation characteristic (ROC) curve was generated, and the area under the curve (AUC) was calculated for each model.

### Gene function enrichment

Gene set variation analysis (GSVA) enrichment of gene ontology (GO) and Kyoto Encyclopedia of Genes and Genomes (KEGG) was performed using the “GSVA” package in R. The GO and KEGG enrichment analysis was conducted using the “clusterProfiler” package in R. This package was also employed for gene set enrichment analysis (GSEA) of the DEGs between high and low NAS groups.

### Immune microenvironment analysis

The “ESTIMATE” package in R was applied to analyze the infiltration ratio of immunocytes and stromal cells and to estimate the immune score and tumor purity within every TCGA, CGGA325, and CGGA693 sample. The immune cell components in the microenvironment were analyzed using the CIBERSORT algorithm.

### RNA velocity and inter-cell communication analysis

RNA velocity was analyzed using the “scVelo” and “velocyto” packages in Python 3.8.8. This analysis revealed the evolutionary pathways of tumor cells based on RNA velocity. Intercellular communication was analyzed using the “celltalker” package in R, and differential ligand-receptor pairs were extracted.

### Transcription factors analysis

For the RNA sequencing (RNA-seq) data, Expression2Kinases (X2K, https://maayanlab.cloud/X2K/) was employed to compare upstream transcription factors of DEGs between low and high NAS groups in TCGA, CGGA325, and CGGA693 datasets. For the scSeq data, we applied pySCENIC algorithm to construct the transcription factors’ regulatory network. The human transcription factors data were downloaded from cisTarget databases (https://resources.aertslab.org/cistarget/) for the network construction. The activation of transcription factors was estimated using the “AUCell” package in R.

### Least absolute shrinkage and selection operator (Lasso) analysis

For the simplify of NAS calculation, lasso analysis was performed with R package “glmnet”. The datasets of TCGA, CGGA325 and CGGA693 were merged into one by their common DEGs. For the parameters of “glmnet” function, we used family = "cox", alpha = 1, nlambda = 100.

### TCR-neoantigen peptide pairs identification

The TCR clonotypes were clustered into patterns using the GLIPH2 algorithm (http://50.255.35.37:8080/), and the binding probability between selected TCR clonotypes and potential neoantigen peptides was analyzed using the DLpTCR algorithm (http://jianglab.org.cn/DLpTCR/). Differences in the clonotypes before and after vaccination were analyzed using the “scRepertoire” package in R.

### Patients and tissue specimens

For immunohistochemistry staining, the paraffin-embedded glioma tissues of WHO grade II (n = 3), III (n = 3) and IV (n = 3) were acquired from glioma patients who underwent surgery in Department of Neurosurgery, Qilu Hospital of Shandong University. And the normal brain tissues (n = 3) were from craniocerebral trauma patients whose normal brain must be partially resected for decompression. And for the quantitative real-time PCR, the frozen glioma tissues for RNA extraction of WHO grade II (n = 4) and IV (n = 4) were also acquired from glioma patients in Department of Neurosurgery, Qilu Hospital of Shandong University.

### Immunohistochemistry (IHC) staining

The formalin-fixed and paraffin-embedded tissues were sectioned into 4 μm slices. Antigen retrieval was conducted in boiled sodium citrate buffer (pH 6.0). Then endogenous horseradish peroxidase was blocked with 3% H_2_O_2_ and the tissues were blocked with 10% normal goat serum. Then the slides were incubated with primary antibodies targeting TMSB10 (Elabscience #E-ab-15878, 1:100 dilution), vimentin (VIM) (Cell Signaling #5741S, 1:200 dilution) and PD-L1 (Invitrogen #14–5982-82, 1:100 dilution). Then target protein was visualized with DAB with standard protocols. The cell nuclei were stained with hematoxylin. Then images were obtained with an Olympus inverted microscope.

### Cell lines and cell culture

LN229, U118MG, A172, U251MG and Jurkat cells were obtained from Culture Collection of the Chinese Academy of Sciences. The GBM#P3, GBM#BG5 and GBM#BG7 were patient-derived glioblastoma stem-like cells isolated from glioblastoma specimens and were functionally characterized [39, 40]. The LN229, U118MG, A172, U251MG glioma cell lines were cultured in Dulbecco’s modified Eagle medium (Macgene, #CM10017) with 10% fetal bovine serum (FBS). The Jurkat cell was cultured in RPMI-1640 (Macgene, #CM10041) with 10% FBS. The GBM#P3, GBM#BG5 and GBM#BG7 were cultured in Neurobasal™ medium (Gibco/Thermo Fisher Scientific, # 21,103,049) supplemented with 10 ng/ml basic fibroblast growth factor (bFGF; PeproTech, #100-18B), 20 ng/ml epidermal growth factor (EGF; Thermo Fisher Scientific, # PHG0311L) and 2% B-27™ Neuro Mix (Thermo Fisher Scientific, # A1895601).

### Quantitative real-time PCR

Total RNA from cells or tissues was extracted with the RNA-Quick Purification Kit (#RN001, ESscience Biotech). 1000 ng of total RNA was synthesized into cDNA with Hifair^®^ III 1st Strand cDNA Synthesis SuperMix (Yeasen Biotechnology, #11141ES10). Then Hieff^®^ qPCR SYBR Green Master Mix (Yeasen Biotechnology, #11201ES03) was applied for amplification in the quantitative real-time PCR. The volume of reaction mixture was 10 μl and the reaction procedure was set according to the manufacturer’s two-step protocol. The primer sequences were listed in Additional file 14: Table S3.

### Cell counting kit-8 (CCK-8) assay

The Jurkat cells were seeded into 96-well plates at a density of 1 × 10^5^ per well with BAPTA-AM at 0, 10, 20 and 40 μM. Then CCK-8 assay was conducted following manufacturer’s protocol (Yeasen Biotechnology, #40203ES76) at the time of 0, 24, 48 h after seeding. The OD values were measured at the wave length of 450 nm.

### Calcium colorimetric assay

Jurkat cells were seeded into 6-well plate at a density of 5 × 10^5^ per well and were treated with BAPTA-AM at 0, 10, 20 and 40 μM for 48 h. Then calcium colorimetric assay was performed with Calcium Colorimetric Assay Kit (Beyotime Biotechnology, #S1063S) following manufacturer’s protocol. Briefly, after counting under microscope, cells were collected by 600 g centrifugation of 5 min. Then cells were treated with lysis buffer and the lysate were centrifugated again at 12000 g for 5 min. Then the supernatant was extracted and used for calcium colorimetric assay to detect calcium concentration. Then calcium mass per million cells was calculated.

### Co-culture and ELISA assay

Jurkat cells were seeded into 6-well plate at a density of 5 × 10^5^ per well. And they were activated by adding 2 μg/ml soluble anti-CD3 (Proteintech, #60,181–1-Ig) and 1 μg/ml soluble anti-CD28 (Proteintech, #65,099–1-Ig) for 24 h, as previously described [41]. Then activated Jurkat cells were seeded into 24-well plate at a density of 1 × 10^5^ per well and BAPTA-AM was added to activated Jurkat cells at 0, 10, 20 and 40 μM for 24 h. At the same day of BAPTA-AM treatment, the LN229 cells were seeded into 96-well plates at a density of 3 × 10^3^ per well. After BAPTA-AM treatment, Jurkat cells were added to LN229 cells at 2 × 10^3^ per well. After 48 h co-culture, the supernatant was extracted for ELISA assay detecting IFN-γ (Proteintech, #KE00146). The remaining LN229 cells were used for CCK-8 assay.

### Statistical analysis

Shapiro-Wilk test was applied to normality test. The Mann-Whitney test was used to compare two groups of data that did not subject to normal distribution and Student’s t-test was employed to compare two groups of data that did. And the ANOVA test was employed to conduct multiple comparisons. The log-rank test was used to determine the overall survival outcome, and the Spearman test was used to analyze the correlations between two sets of data. Analyses were conducted using R (version 4.1.3), Python (version 3.8.8), and GraphPad Prism (version 8.3.0).

## Results

### Non-m6A-related neoantigen-coding lncRNAs were identified in glioma patients in TCGA dataset

The overall workflow of this study was manifested in Additional file 1: Fig. S1A. First, the expression levels of non-m6A-related regulators in TCGA dataset were collected. In accordance with a previous study [35], the non-m6A modifications were divided into the following three classes according to their levels: 1) high to intermediate levels of modifications with hundreds to thousands of modification sites (Ψ and m5C); 2) ultra-low levels of modifications with few modification sites (m1A); and 3) unknown levels of modification that require further confirmation (like N4-acetylcytidine [ac4C], 2'-O-methylation [Nm], and 7-Methylguanosine [m7G]). Next, modifications in the first two classes, including Ψ, m5C, and m1A, were selected as the main non-m6A modifications to be investigated. The regulators involved in Ψ, m5C, and m1A modification are listed in Additional file15: Table S4 [36–38]. We identified the non-m6A-correlated lncRNA in TCGA dataset (Additional file 1: Fig. S1B) with the function “cor.test” in R. The p value threshold was set to 0.001 and |R| threshold was set to 0.4 (Additional file 16: Table S5). And the correlation network was constructed with “igraph” package in R. Then lncRNAs that significantly correlated with glioma patients' overall survival time were selected (Additional file 1: Fig. S1C) using uniCox analysis. According to TransLnc, potential peptide-coding lncRNAs in which peptides could be presented by MHC I were determined as MHC I is the main contributor of endogenous antigens, including tumor-derived antigens. A total of 13 lncRNAs were discovered, 5 of which had an HR of greater than 1 and 8 of which had an HR of less than 1. These 13 lncRNAs were significantly upregulated in the glioma group compared to the normal group (Additional file 1: Fig. S1D).

### Non-m6A modification is activated in high grade gliomas and NAS predicts glioma patients' prognosis

**Fig. 1:**
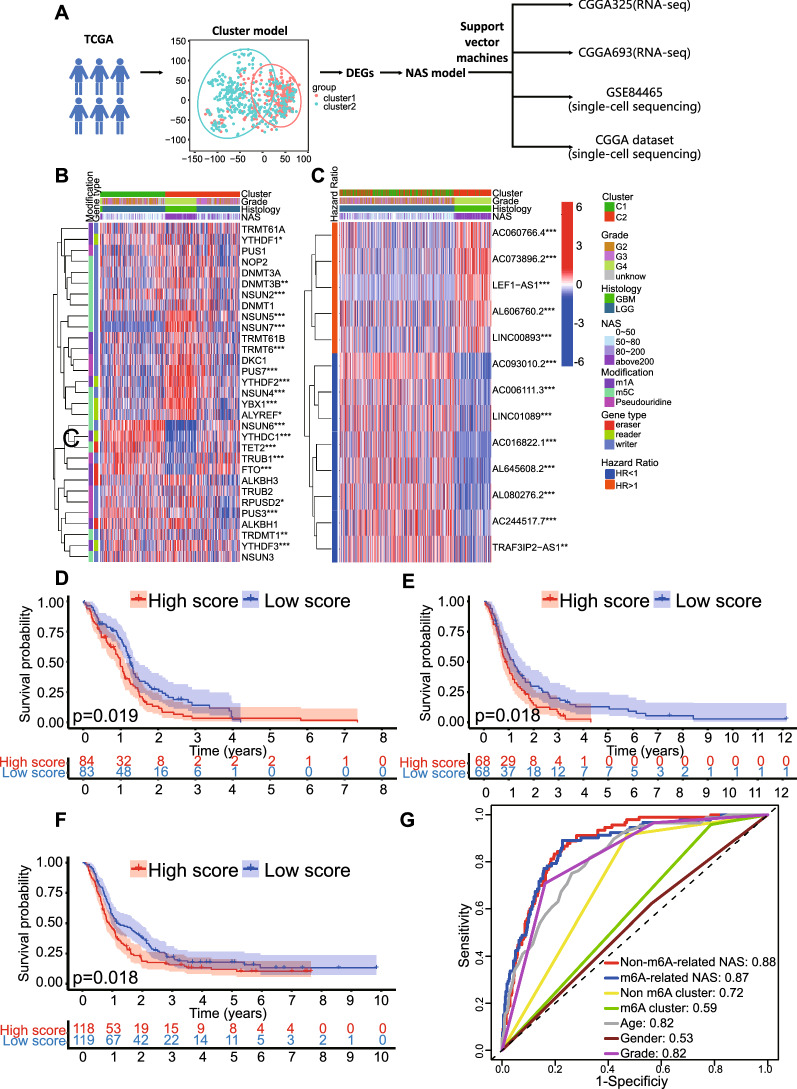
The construction and comparison of NAS models. **A** Flow chart of the process of NAS model construction. **B** non-m6A-related regulators’ expression and corresponding clinical and genetic characteristics based on NAS were shown in heatmap. **C** neoantigen-coding prognosis-related lncRNAs’ expression and corresponding clinical and genetic characteristics based on NAS were shown in heatmap. **D**–**F** Overall survival analysis based on the non-m6A-related NAS in TCGA, CGGA325, CGGA693, respectively. **G** Comparison of non-m6A-related NAS models and other prognostic models with ROC curves in TCGA dataset

The process of establishing the NAS model is illustrated in Fig. 1A andAdditional file 1: Fig. S1A. Consensus clustering was conducted based on the expression of non-m6A-related neoantigen-coding lncRNAs. The glioma samples from TCGA were divided into two clusters to acquire the highest relability in every cluster (Additional file 2: Fig. S2A), and principal component analysis (PCA) plotting revealed differences in the distribution between cluster 1 and cluster 2 (Additional file 2: Fig. S2B). We identified the expression of selected non-m6A regulators in these two clusters, and results indicate that most of the writers and readers were upregulated in cluster 2 (Fig. 1B). For the erasers, the FTO and TET2 were significantly elevated in cluster 1 while levels of ALKBH1 and ALKBH3 were not significantly different between the two clusters (Fig. 1B). This suggests an enhanced non-m6A modification state in cluster 2 compared to cluster 1. In cluster 2, the lncRNAs with an HR greater than 1 were of a significantly higher expression level than those in cluster 1, while those with an HR less than 1 were of a significantly lower expression level (Fig. 1C). Then SVM algorithm was used to learn the gene expression features of cluster1 and cluster2 and to reproduce the clustering model in validation datasets. Similar results were observed in the CGGA325 dataset (Additional file 2: Fig. S2C), while, in the CGGA693 dataset, only a few genes were significantly differently expressed between the two clusters (Additional file 2: Fig. S2D). Furthermore, the cluster model predicted the prognosis efficiently in all gliomas and LGG. In the model, cluster 2 manifested a significantly worse prognosis than cluster 1, while in terms of GBM, there was no significant difference between the clusters (Additional file 3: Fig. S3). However, in ROC analysis, the prognostic efficacy of the cluster model was less than satisfactory in that the AUC value of the non-m6A-related neoantigen cluster model was 0.72 (Fig. 1G), which was even less than the that of age (AUC = 0.82) and grade (AUC = 0.82).

We hypothesized that the accuracy of cluster model was limited by its binary classification of TCGA samples. To improve the accuracy of the non-m6A-related neoantigen cluster model, an NAS model was developed based on the DEGs between cluster 1 and cluster 2 according to a previous study [34], as discussed in the “Methods” section. As mentioned above, the formula included gene expression levels and their weight produced by PCA, so it provided a quantified parameter that was related to the expression of genes with HR > 1 and HR < 1. According to our analysis on clustering model, cluster2 showed worse prognosis than cluster1, and all DEGs of HR > 1with significant prognostic effects elevate in cluster2 compared to cluster1. HR > 1 also indicates that high expression of a gene is associated with worse prognosis. Also, all DEGs of HR < 1 with significant prognostic effects downregulate in cluster2. This result is shown in venn diagram (Additional file 2: Fig. S2E). Therefore, the formula basically reflects the similarity degree between the gene expression pattern of an RNA-seq sample and cluster2. The NAS model distinguished glioma subtypes at different levels more precisely than the cluster model and was highly correlated with the expression of selected regulators or lncRNAs (Fig. 1B, C). Similar results were obtained when NASs were calculated in the CGGA325 and CGGA693 datasets (Additional file 2: Fig. S2C, D). Moreover, the NAS model performed well when predicting the prognosis of GBM patients in TCGA (Fig. 1D), CGGA325 (Fig. 1E), and CGGA694 (Fig. 1F) datasets in that the high NAS group presented shorter average survival time. Similar results were found in all glioma and LGG groups in these three datasets (Additional file 4: Fig. S4). The AUC value of the non-m6A-related NAS model was 0.88, which was much higher than in the cluster model, as were the values for age and grade (Fig. 1G).

To identify the difference between the non-m6A-related NAS model and m6A-related NAS model, the m6A-related NAS model was constructed in a similar manner (Additional file 5: Fig. S5). The AUC value of the m6A clustering model was 0.59, while that of the NAS model was 0.87 (Fig. 1G). Then data of all 3 datasets (TCGA, CGGA325 and CGGA693) were merged and AUC values of all models were calculated again. The results indicated that AUC value of non-m6A-related NAS model was 0.76 (Additional file 1: Fig. S1E), larger than m6A-related NAS model (AUC = 0.66). It suggested the non-m6A-related model had better prognostic accuracy than the m6A-related model in all three datasets. Therefore, the non-m6A-related NAS model was selected for further research.

### High NAS are correlated with aggressive subtypes of glioma

**Fig. 2:**
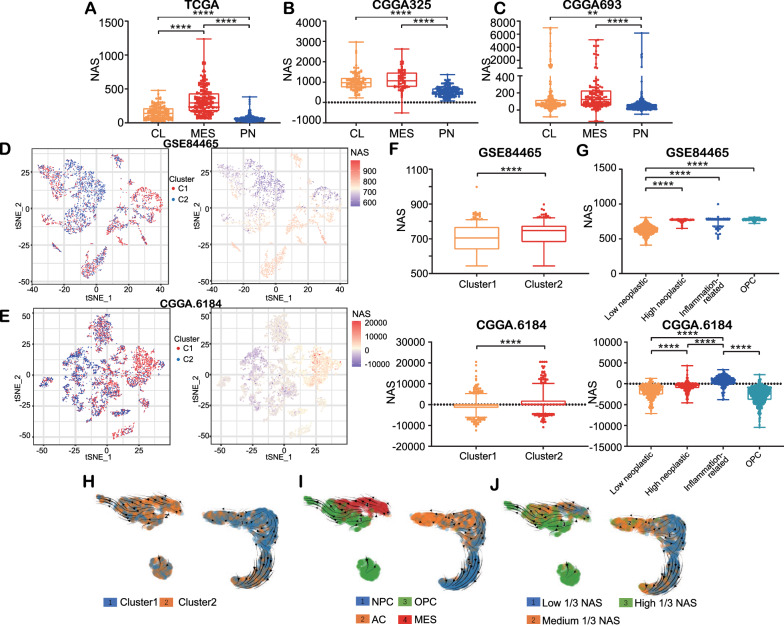
The NAS is positively correlated with glioma aggressiveness. **A**–**C** Distribution of NAS in samples grouped by Verhaak classification in TCGA, CGGA325, CGGA693, respectively. **D**, **E** Distribution of clusters and NAS in GSE84465 and CGGA dataset, respectively. The cells are displayed by tSNE reduction. **F** The NAS of cluster1 and cluster2 in GSE84465 and CGGA dataset, respectively. **G** The NAS of four cell types, low neoplastic, high neoplastic, inflammation-related glioma cells and OPC in GSE84465 and CGGA scSeq datasets, respectively. **H**–**J** The RNA velocity of GSE84465 displayed by clusters, subtypes and NAS, respectively

In order to identify the relationship between NAS and glioma subtype, non-negative matrix factorization (NMF) clustering was used to divide the samples into three clusters according to Verhaak classification, as described in previous literature [33]. The results showed that, of the three subtypes, the mesenchymal (MES) subtype had the highest average NAS, and the proneural (PN) subtype showed the lowest average NAS in all three datasets (Fig. 2A–C). The MES subtype was the most aggressive subtype and was associated with poor survival outcomes, while the PN subtype displayed the lowest levels of aggressiveness [33]. Therefore, the NAS was positively correlated with the aggressiveness of gliomas, as predicted by the Verhaak classification using bulk sequencing data.

The scSeq data of glioma cells in the GSE84465 dataset were clustered and annotated (Additional file 6: Fig. S6A) following a previous study [34] with a slight modification, and the marker genes in every cluster were identified (Additional file 6: Fig. S6C). Results were reproduced in the CGGA scSeq dataset using the same procedure (Additional file 6: Fig. S6B, D). Moreover, the cells were also divided into two clusters using SVM. Based on the cluster model, the DEGs were explored using the “DEsingle” package in R (Additional file 17: Table S6), and the NAS was calculated. Cluster 2 overlapped extensively with cells with a relatively high NAS in the t-distributed stochastic neighbor embedding (t-SNE) reduction plot (Fig. 2D, E), and the Mann–Whitney test confirmed these results (Fig. 2F). Additionally, we also found that the low neoplastic cells showed a significantly lower NAS than the high neoplastic and inflammation-related glioma cells in both the GSE84465 and CGGA datasets (Fig. 2G). We then classified the cells in the GSE84465 dataset into four previously established molecular subtypes on a single cell level based on existing literature [34, 42]. The RNA velocity of glioma cells in the GSE84465 dataset was then calculated by analyzing the abundance of spliced and unspliced RNA so as to profile the evolutionary processes of glioma cells. Most of the cells in cluster 2 were at the end of their revolutionary processes (Fig. 2H). Meanwhile, the MES and oligodendrocyte-progenitor-like (OPC) subtypes were located at the end of the revolutionary pathway (Fig. 2I), indicating that most glioma cells become more aggressive over time. NAS increased as the glioma cells developed (Fig. 2J), indicating that NAS reflects the stage of glioma cell revolution. These results were also confirmed through pseudo-time analysis (Additional file 6: Fig. S6I). Additionally, cell trajectory analysis showed that OPCs and high neoplastic glioma cells were at the apex of cell trajectory, while low neoplastic cells were upstream of cell trajectory both in the GSE84465 (Additional file 6: Fig. S6E, G) and CGGA datasets (Additional file 6: Fig. S6F, H). The fact that high neoplastic glioma cells had higher NASs than low neoplastic ones suggests that glioma cells develop to be more aggressive when this development is accompanied by higher NAS. Together, these data indicate a positive correlation between NAS and glioma aggression.

### NAS model is associated with antigen-presenting and T-cell-related pathways

**Fig. 3:**
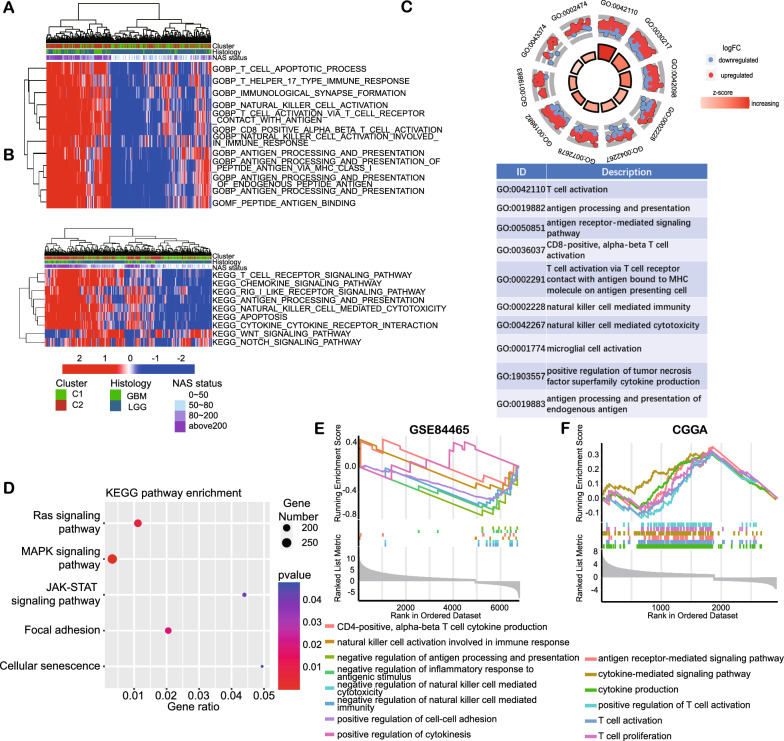
Biofunction enrichment between low or high NAS groups based on TCGA and scSeq datasets. **A**, **B** The GSVA analysis of GO and KEGG pathways of TCGA samples is illustrated by heatmap and clinical features. **C**, **D** GO and KEGG enrichment analysis based on DEGs between low and high NAS groups in TCGA dataset. **E**, **F** GSEA enrichment analysis based on DEGs between low and high NAS groups in GSE84465 and CGGA scSeq datasets, respectively

To identify the biofunctions involved in differences in NAS levels, we conducted enrichment analysis with the DEGs of TCGA samples with low or high NAS. GSVA enrichment analysis revealed that samples with a high NAS showed a higher enrichment score in T-cell-mediated immunity, NK-cell-mediated cytotoxicity, and antigen processing and presentation in both the GO and KEGG pathways (Fig. 3A, B). GO enrichment analysis had very similar results in terms of T-cell-related functions, antigen processing, and presentation (Fig. 3C) as well as the significant pathways enriched in the KEGG database, including immune-related pathways, such as NK-cell-mediated cytotoxicity, the chemokine signaling pathway, and antigen processing and presentation (Fig. 3D). GSEA enrichment analysis of the scSeq data revealed that anti-tumor immunity factors, such as T cell cytokine production and NK-cell activation, were elevated in the high NAS group (Fig. 3E). However, cell-cell adhesion was downregulated in this dataset. Analysis of the scSeq data in the CGGA dataset also showed facilitated T-cell-related functions in the high NAS group (Fig. 3F). Taken together, NAS and the non-m6A-related neoantigen-coding lncRNAs were associated with T-cell-related immunity and antigen processing and presentation.

### Higher NAS gliomas are correlated with higher levels of immune infiltration

**Fig. 4:**
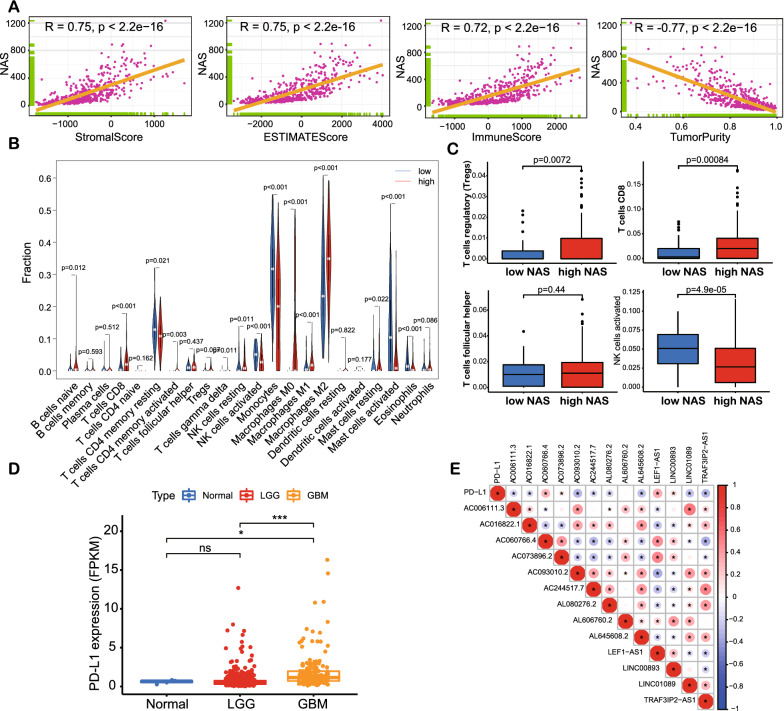
The immune landscape based on bulk RNA-seq data of TCGA. **A** Correlations between Stromal score, ESTIMATE score, Immune score, tumor purity and NAS. **B** Infiltration ratio of all immunocytes in low and high NAS groups analyzed by CIBERSORT. **C** Infiltration ratio of Treg, CD8 + T cells, T helper and activated NK cells in low and high NAS groups based on CIBERSORT analysis. **D** The expression of PD-L1 in normal, LGG and GBM samples. **E** The correlations between expression of PD-L1 and non-m6A-related neoantigen-coding lncRNAs

To determine the relationship between NAS and immune infiltration, the “ESTIMATE” package in R was employed to assess immune infiltration in the samples from TCGA dataset. NAS was positively correlated with immune score and negatively correlated with tumor purity (Fig. 4A), suggesting that a higher NAS implies higher levels of immune infiltration. Analysis of the samples from the CGGA325 and CGGA693 datasets revealed similar trends (Additional file 7: Fig. S7A, B). Following this, we used CIBERSORT to profile changes of infiltrated immune cells in detail, with results indicating that, although the proportion of CD8 + T cells and T helper cells was elevated, the proportion of regulatory T (Treg) cells increased while the proportion of activated NK cells declined (Fig. 4B, C). Similar results were observed in the samples from the CGGA325 and CGGA693 datasets; CD8 + T cells and the proportion of Treg cells were elevated in the high NAS group (Additional file 7: Fig. S7C–F). Given that a higher NAS suggests poorer survival outcomes and more aggressive tumors, we analyzed the possible mechanisms underlying higher immune infiltration that does not suppress glioma cells. The expression of PD-L1 was elevated when the glioma grade rose, indicating higher immunosuppression (Fig. 4D). It was also found that the expression of four lncRNAs (i.e., AC060766.4, AC0738962, LEF-AS1, and LINC00893) were positively correlated with PD-L1 (Fig. 4E). These four lncRNAs were included in the five lncRNAs with hazard ratios greater than 1 (Additional file 1: Fig. S1B). Thus, it was clear that immune infiltration was higher in the high NAS group than in the low NAS group and that increased PD-L1 expression might suppress the activation of T-cell-mediated immunity.

**Fig. 5:**
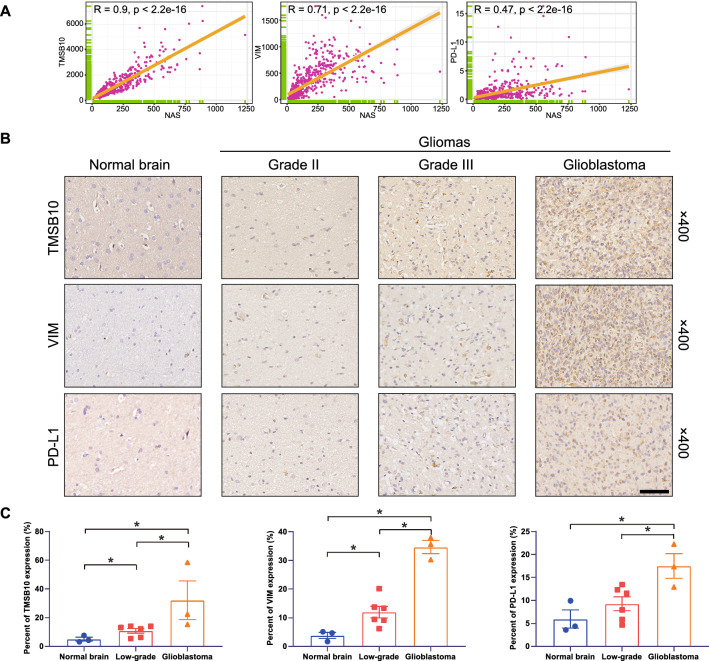
IHC results of NAS-related genes (TMSB10 and VIM) and PD-L1. **A** Correlations between NAS and expression levels of TMSB10, VIM and PD-L1, respectively. **B** Representative IHC images of TMSB10 B, VIM C and PD-L1 D, respectively. Normal brain tissues (n = 3) and glioma tissues of WHO grade II (n = 3), III (n = 3) and IV (n = 3) were used. **C** Positive ratios of TMSB10, VIM and PD-L1 in IHC results, respectively. Bar = 50 μm

The expressions of NAS-related genes and PD-L1 were detected with IHC. In NAS, PC1 + PC2 was the main index influencing final results. TMSB10 was the gene with the highest PC1 + PC2 value (Additional file 7: Fig. S7G). VIM was in the top 10 list of PC1 + PC2 value, and it was also reported to be a marker of tumor invasiveness [43] and immunosuppression [44]. The correlations between NAS and expression of TMSB10, VIM and PD-L1 were checked (Fig. 5A). It was shown that NAS was significantly correlated with expression of TMSB10 (R = 0.9), VIM (R = 0.71) and PD-L1 (R = 0.47). Then we detected expression of TMSB10, VIM and PD-L1 with IHC. It was found that gliomas of more advanced grades exhibited higher TMSB10, VIM and PD-L1 levels (Fig. 5B, C). These results suggest genes positively correlated with NAS and PD-L1 are expressed more in higher grade gliomas, and it also indicates higher NAS might be associated with more PD-L1 expression.

### Abnormal expression of T-cell positive regulators especially calcium-related genes in high NAS group might lead to T cell malfunction

**Fig. 6:**
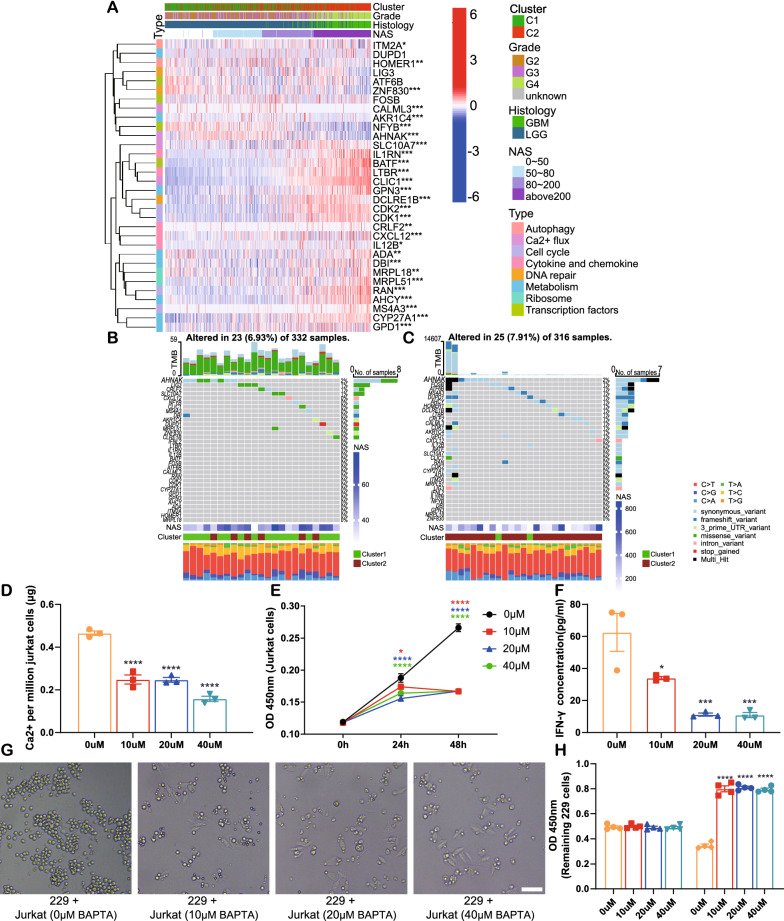
Expression of positive regulators of T cells and their mutations in TCGA dataset. **A** Expression of positive regulators of T cells illustrated by heatmap based on clinical features and NAS. **B**, **C** Single nucleotide variations of positive regulators of T cells in low (**B**) or high (**C**) NAS groups in TCGA dataset, respectively. **D** The calcium mass per million Jurkat cells after BAPTA-AM treatment at the concentration of 0, 10, 20 and 40 μM. **E** The cell viability of Jurkat cells after BAPTA-AM treatment. **F** IFN-γ concentrations in the co-culture medium. **G**, **H** The microscopic images (**G**) and viability of remaining LN229 cells (**H**) of co-culture system after 48 h. Bar = 100 μm

We found conflicting results in that a high NAS is associated with more immune infiltration, but it also predicts a worse prognosis; this suggests that the infiltrating immunocytes do not suppress glioma cell growth. We analyzed the functions of T cells, which are the direct executioners of anti-tumor immunity. A recent study screened regulators that could positively regulate the function of T cells, and 33 genes with varying functions were identified [45]. Therefore, it was necessary to analyze the possibility that abnormal expression and mutation patterns of these 33 genes resulted in T cell malfunctions. Genes related to Ca^2+^ flux (AHNAK and CALML3), DNA repair (ZNF830), and autophagy (HOMER1) were significantly downregulated in the high NAS group of the sample from TCGA dataset (Fig. 6A). Similar results were achieved by analyses of the CGGA325 and CGGA693 datasets (Additional file 8: Fig. S8A, B). The other regulators were upregulated in the high NAS group.

Moreover, the SNV data in TCGA sample showed that the TMB of these 33 genes was higher in the high NAS group than in the low NAS group without statistical significance (Additional file 18: Table S7) and that AHNAK contributed most of the mutations to high and low NAS groups (Fig. 6B, C). Similarly, the samples in cluster 2 showed more mutation burdens related to these 33 genes than those in cluster 1 (Additional file 8: Fig. S8C, D). T cells of high-grade gliomas might be of interference with Ca^2+^ flux, DNA repair, autophagy, and the aldo–keto metabolism. Among these gene functions, Ca^2+^ flux mediated by AHNAK was highlighted as this gene displayed the most mutations among the 33 genes studied. Then we tried to validate the role of Ca^2+^ in T cell function by in vitro functional assay. The intracellular Ca^2+^ chelator BAPTA-AM [46] was applied to Jurkat cells at the concentration of 0, 10, 20 and 40 μM for 48 h. Then intracellular calcium was detected and it significantly decreased in BAPTA-AM groups (Fig. 6D). Jurkat cell proliferation was also significantly inhibited by BAPTA-AM treatment (Fig. 6E). For the co-culture assay, the workflow was shown in Additional file 8: Fig S8E. Briefly, the Jurkat cell were activated with CD3/CD28 antibodies for 24 h according to a previous study [41]. After another 24 h’ treatment with BAPTA, activated Jurkat cells were co-cultured with LN229 cells. 48 h later, the IFN-γ in culture medium, which was regarded as a marker of T cell activation [41], was detected by ELISA. The results suggested that IFN-γ secretion was also inhibited in BAPTA-AM groups (Fig. 6F). The increase of remaining LN229 cells in BAPTA-AM groups also indicated suppressed function of activated Jurkat cells (Fig. 6G, H). Taken together, in high NAS samples, abnormal Ca^2+^ flux might play an essential role in the failure of T cell-mediated glioma growth suppression.

### High NAS gliomas are associated with transcription factors involved in stemness

**Fig. 7:**
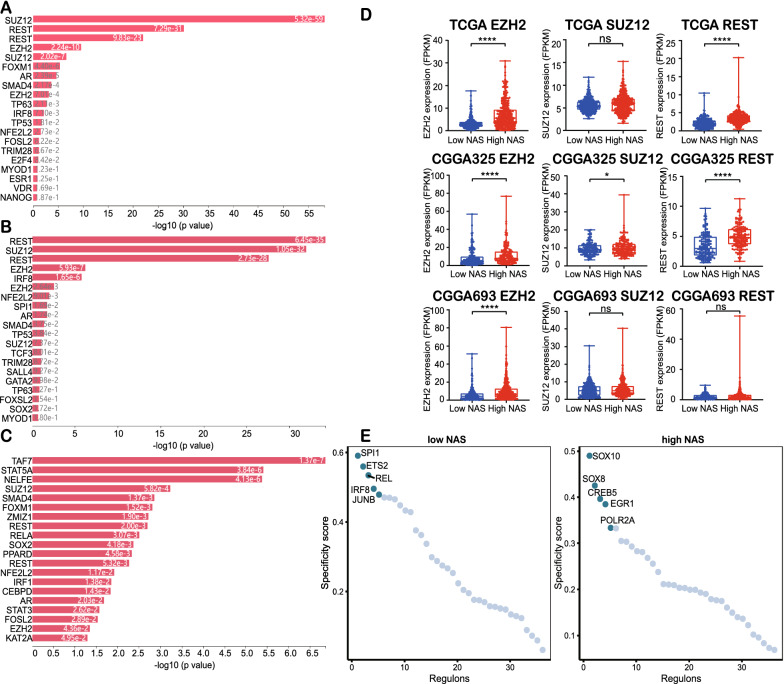
Transcription factors analysis revealed enhanced stemness-related transcription factors’ activities in high NAS groups. **A-C** The top 20 transcription factors enriched by X2K in TCGA **A**, CGGA325 **B**, CGGA693 **C** datasets, respectively. **D** The expression levels of EZH2, SUZ12 and REST in TCGA, CGGA325 and CGGA693 datasets, respectively. **E** The top 5 activated transcription factors analyzed by pySCENIC in low and high NAS groups in GSE84465 dataset

Upstream transcription factors were also important for elucidation of potential mechanisms in NAS changes. The X2K was employed in order to determine the upstream regulatory transcription factor networks in TCGA, CGGA325, and CGGA693 datasets. The DEGs of the low and high NAS groups were imported into X2K, after which the top 20 upstream regulatory transcription factors in TCGA (Fig. 7A), CGGA325, (Fig. 7B), and CGGA693 (Fig. 7C) datasets were obtained. We noticed that some of the top 20 transcription factors were common across all three datasets, such as the SUZ12, REST, EZH2, SMAD4 and AR. The SUZ12 [47], REST [48], and EZH2 [49] genes were reported to contribute to the stemness of cancer cells, and they exhibited higher expression levels in the higher NAS group (Fig. 7D). These results indicate that higher NAS is associated with the facilitation of stemness transcription factors’ expression in RNA-seq data.

In terms of the scSeq data, we applied pySCENIC in the construction of regulatory networks of transcription factors. The differential activated transcription factors in the GSE84465 dataset were calculated (Fig. 7E). The top 5 activated transcription factors were demonstrated, and stemness-related genes, such as SOX10 [50] and SOX8 [51], were activated in the higher NAS cells, while in the lower NAS cells, no such genes were observed in the top 5 activated transcription factors. However, in the CGGA scSeq data, opposite results were obtained (Additional file 9: Fig. S9A). We conducted another validation in dataset GSE129671. Among the five most activated transcription factors, MYC [52] in high NAS group was reported to facilitate cell stemness.

**Fig. 8:**
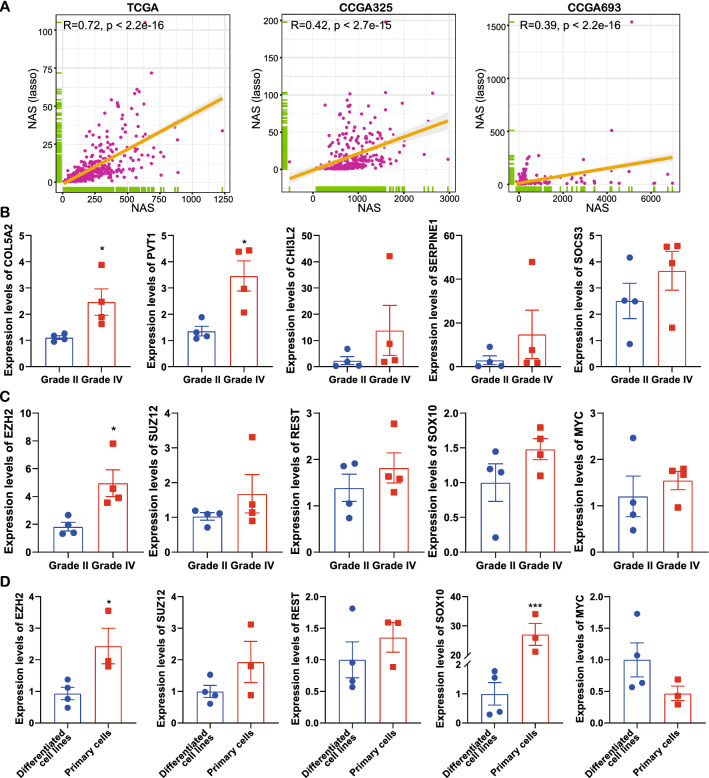
The expression levels of stemness-related genes in glioma specimens and glioma cell lines. **A** The correlation analysis between NAS and lasso-derived-NAS in TCGA, CGGA325 and CGGA693 datasets. **B** Expression of NAS-related genes in glioma specimens. **C** Expression of stemness-related genes in glioma specimens.** D** Expression of stemness-related genes in differentiated and stem-like glioma cell lines

To investigate the stemness-related and NAS-related genes’ expressions in glioma specimens, the lasso analysis was applied to identify major contributors to NAS. By merging TCGA, CGGA325 and CGGA693 datasets, lasso regression identified five genes and a simplified NAS could be calculated as: NAS = 0.22864885*(COL5A2 expression level) + 0.1083532*(PVT1 expression level) + 0.07381116*(CHI3L2 expression level) + 0.03597545*(SERPINE1 expression level) + 0.02452755*(SOCS3 expression level). And the lasso-derived NAS was highly correlated with NAS in all three datasets (Fig. 8A). Then these five genes were detected with qRT-PCR in 4 grade II and 4 grade IV gliomas. The results indicated all five genes elevated in grade IV gliomas and COL5A2 and PVT1 showed statistical significance (Fig. 8B), suggesting higher NAS in grade IV gliomas. And five stemness-related genes identified by analyses above were also detected, including EZH2, SUZ12, REST, SOX10 and MYC. The results showed only EZH2 significantly elevated in grade IV gliomas (Fig. 8C). These five genes were also detected in our patient-derived glioblastoma stem-like cells (GBM#P3, GBM#BG5 and GBM#BG7) and differentiated cells (A172, U118MG, U251MG and LN229). It suggested EZH2 and SOX10 were significantly elevated in stem-like cells (Fig. 8D). These results indicated that higher stemness existed in the higher NAS groups of most samples and datasets. And EZH2 was the most significantly elevated stemness-related gene in our assay.

### Enhanced T cell-glioma cell interactions in high NAS group might be escaped via low interferon-γ receptor (IFNγR) pathway expression

**Fig. 9:**
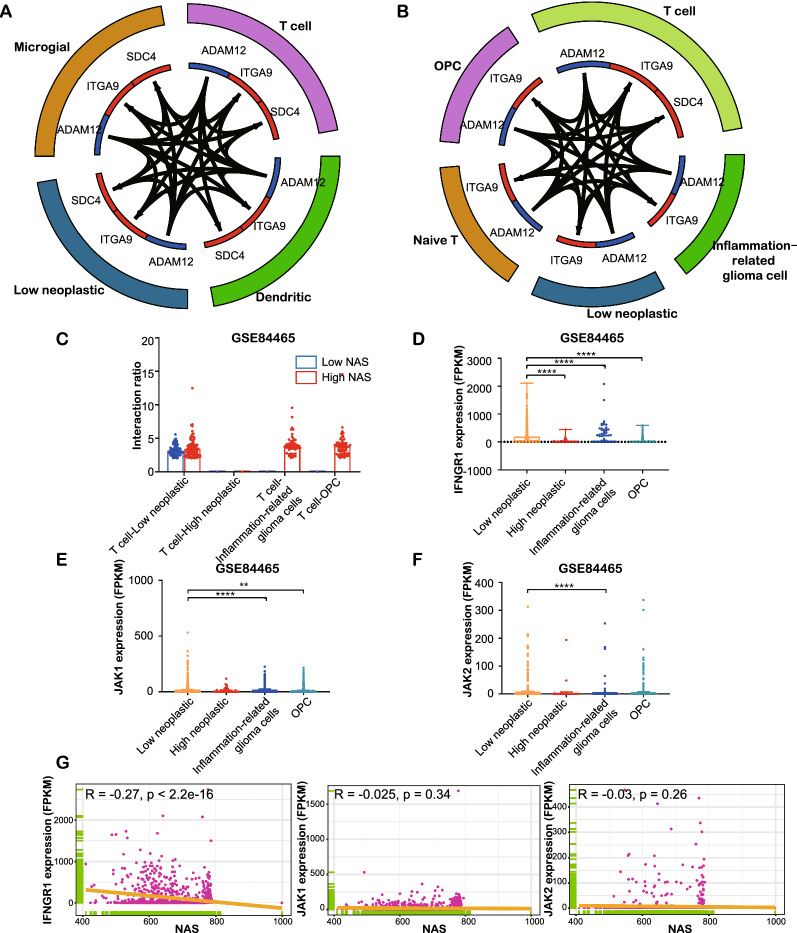
Intercellular communication analysis of single cell RNA-seq based on GSE84465. **A**, **B** Top 30 interactions between cells in low or high NAS groups, respectively. **C** The interaction ratios between T cells and four kinds of glioma cells in single cell RNA-seq of low or high NAS groups. **D**–**F** The expression of IFNGR1, JAK1 and JAK2 in four kinds of glioma cells. **G** The correlations between expression of IFNGR1, JAK1, JAK2 and NAS in glioma cells

We applied the “celltalker” packages in R to analyze inter-cell communication in order to identify potential mechanisms underlying the suppressed function of T cells in the high NAS group. In the low NAS group, low neoplastic cells were active in most significant interactions, interacting with dendritic cells and T cells via the ADAM12 and ITGA9/SDC4 pathways, with both having no points in the total interaction plot (Fig. 9A). Conversely, in the high NAS group, most interactions occurred between T cells and OPC or inflammation-related glioma cells (Fig. 9B, C). The ADAM12 and ITGA9/SDC4 pathways were the two main pathways between these cells. Moreover, significant interactions between T cells and four types of glioma cells were analyzed, and results showed that there were more interactions between T cells and low neoplastic, inflammation-related glioma cells and OPC in the high NAS group than in the low NAS group. Furthermore, in the CGGA dataset, T cells were not involved in most significant interactions in the low NAS group, while they interacted with other immunocytes, such as dendritic and microglial cells, in the high NAS group (Additional file 10: Fig. S10A, B). Additionally, regarding significant interactions, only some of the interactions between T cells and inflammation-related glioma cells in the high NAS group were significant, indicating that inflammation-related glioma cells are involved in T-cell-mediated immunity (Additional file 10: Fig. S10C).

It is important to note that there were no significant interactions between T cells and high neoplastic glioma cells whose NAS was relatively higher than that mentioned above. We deduced that less T cell binding and cell–cell adhesion might be the underlying mechanism. A recent study indicated that the binding of T cells to glioma cells calls for the IFNγR signaling pathway (IFNGR1, JAK1 and JAK2) in which IFNGR1 was found necessary for this kind of cell-cell adhesion [53]. Results showed that expressions of IFNGR1 and JAK1 were significantly higher compared to the other three glioma cell types, but expression of JAK2 was not obviously different (Fig. 9D–F). Furthermore, IFNGR1 was found to be significantly negatively correlated with NAS (Fig. 8G), while JAK1 and JAK2 were not. We also found that the low neoplastic cells showed higher IFNGR1 expression, but JAK1 expression slightly decreased in low neoplastic cells, in the CGGA dataset (Additional file 10: Fig. S10D–F). A significant negative correlation between IFNGR1 and NAS was also found (Additional file 10: Fig. S10G). This suggests an increased interaction between T cells and glioma cells and that high neoplastic cells might escape this interaction by downregulating IFNGR1 to decrease T cell binding.

### TCRs with two patterns are predicted to bind to neoantigens from non-m6A-related lncRNAs

**Fig. 10:**
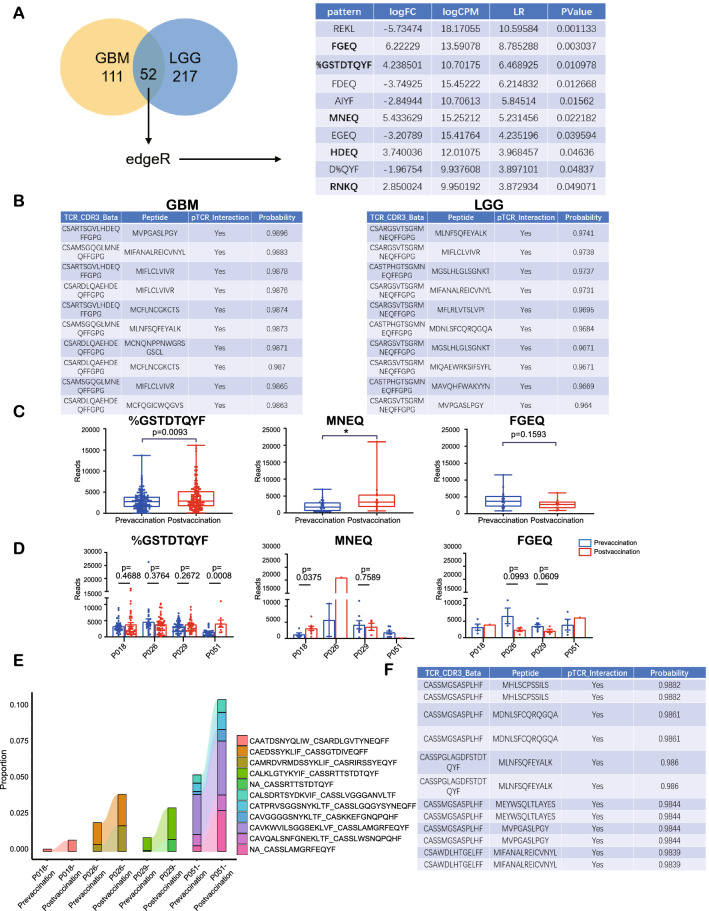
The screening of unique TCR patterns in gliomas compared to normal tissues. **A** The clustering of TCR clonotypes into patterns with GLIPH2 and analysis of patterns with edgeR. **B** The top 10 predicted TCR clonotype-neoantigen pairs by DLpTCR. **C** Total reads of three patterns in prevaccination and postvaccination cells in four patients. **D** Reads of three patterns in prevaccination and postvaccination cells in every patient. **E** The significant expanded TCR clonotypes after vaccination. **F** The top 10 predicted TCR clonotype-neoantigen pairs by DLpTCR with the expanded TCR clonotypes

To determine some possible approaches to glioma therapy based on the non-m6A-related neoantigen model, we screened published TCR sequencing datasets to explore TCR clonotypes that might bind to the peptides coded by the 13 selected non-m6A-related lncRNAs. LGG and GBM samples from GSE79338, a dataset including TCR sequencing data from normal brain tissue, were used to identify unique TCR clonotypes in GBM and LGG. In our search for the MHC-restricted peptide antigens, we clustered TCR clonotypes into complementarity determining region 3 (CDR3) patterns using the GLIPH2 algorithm, and we identified many unique TCR CDR3 patterns in the GBM and LGG samples that could not be found in normal tissues. From these TCR CDR3 patterns, we extracted 52 common patterns in the LGG and GBM samples (Fig. 10A, Additional file 19: Table S8). By comparing the frequencies of the patterns in GBM to those in LGG using the “edgeR” package in R, we identified the 10 patterns that differed the most between the two groups (Fig. 10A). Five of these amino acid patterns, FGEQ, %GSTDTQYF, MNEQ, HDEQ, and RNKQ, were upregulated in the GBM patterns, thereby implying that these patterns might widely exist in glioma patients and that could be related with the progression of gliomas.

Next, we applied the DLpTCR algorithm to assess the possibility of recognition and binding between the MHC I-presenting peptides that may be encoded by the 13 selected lncRNAs and the TCR clonotypes of the 5 selected patterns in the GBM or LGG samples described above. MNEQ and HDEQ were the patterns with the greatest binding probabilities (Fig. 10B).

The scTCR data from the GSE188620 dataset of glioma tissues from four glioma patients before and after tumor-cell lysate vaccination were examined in order to explore the 5 selected patterns. Unique clonotypes were found in the after-vaccination samples (Additional file 11: Fig. S11A-D). However, for the 5 selected patterns, while HDEQ and RNKQ showed extremely low expressions, the other three patterns’ expressions were significantly higher. Furthermore, the total expressions of %GSTDTQYF and MNEQ were significantly elevated in samples after vaccination, which were accompanied by a slight, statistically insignificant decrease in FGEQ (Fig. 10C). Expressions of %GSTDTQYF were elevated in patients 1, 3, and 4, but statistical significance was only found in patient 4 (Fig. 10D). Patients 1 and 2 showed elevated MNEQ expressions (Fig. 10D). We also identified the clonotypes that expanded after vaccination (Fig. 10E), which may include neoantigen-reactive TCR clonotypes. The binding possibility of the expanded TCR clonotypes and peptides was also determined, with 44 TCR-peptide pairs having a binding probability over 0.98; this suggested high binding possibilities between potential neoantigen-reactive TCR clonotypes and selected peptides. In summary, we determined that the peptides that might be encoded by the 13 selected lncRNAs show high binding probabilities with potential neoantigen-reactive TCR clonotypes whose patterns widely exist in glioma tissues. This provides a promising foundation for future CAR-T therapy research.

## Discussion

In this study, we investigated neoantigen-coding lncRNAs using the TransLnc database, and we found that neoantigen-coding lncRNAs related to non-m6A modifications, including pseudouridine, m5C, and m1A, have the potential to play a role in the prediction of glioma patients’ prognoses. The cluster model that was based on the 13 selected non-m6A-related neoantigen-coding lncRNAs could predict the prognosis in all gliomas and LGG. Furthermore, the NAS that was based on the cluster model predicted prognosis better in both GBM and LGG. The NASs based on the non-m6A-related neoantigen-coding lncRNAs together with cluster models were more accurate than those based on the m6A-related models. Moreover, a higher NAS indicates more aggressive gliomas, both at the tumor level and at the cellular level. Enrichment analysis suggested that immune pathways, including endogenous antigen processing and presentation as well as T-cell-mediated immunity, were enriched in high NAS samples. Moreover, NAS was positively correlated with immune infiltration, including CD8 T cell infiltration, but high NAS gliomas also exhibited more PD-L1 expression suggesting an immunosuppression environment, which is consistent with the fact that a higher NAS predicts worse survival outcomes. Further analysis of the immunosuppression of microenvironment T cells revealed that several positive regulators of T cells downregulate functions, including Ca^2+^ flux and DNA repair. The Ca^2+^ flux-related gene AHNAK also manifested most of the mutations present in TCGA dataset. Transcription factor analysis indicated that the expression of stemness-related transcription factors was elevated in the high NAS group compared to the low NAS group. Additionally, cell communication analysis confirmed that the high NAS group showed more inter-cell communication between immune cells and inflammation-related glioma cells and that high neoplastic glioma cells might escape T cell binding by downregulating IFNGR1. Finally, we identified several unique TCR CDR3 patterns that widely exist in glioma tissues, two of which (%GSTDTQYF and MNEQ) were significantly elevated in glioma tissues after tumor-cell lysate vaccination. And increased levels of these two neoantigen-reactive TCR patterns were found in high NAS gliomas, suggesting NAS model was also correlated with neoantigen response. Therefore, non-m6A-related neoantigen-coding lncRNAs play an essential role in neoantigen-related immune microenvironment of gliomas, which is a conclusion that provides a potential avenue for future CAR-T therapy, which could have wide targetability among glioma patients. Our non-m6A-related NAS model exhibited higher prognostic efficacy in TCGA dataset than m6A-related NAS model, m6A/non-m6A clustering model, age, gender and grade (Fig. 1G). In all three RNA-seq databsets, non-m6A-related NAS model also showed obviously better prognostic effect than m6A-related NAS model and was close to WHO grade model (Additional file 1: Fig. S1E). Moreover, the non-m6A-related NAS model was also highly associated with aggressive subtypes in both RNA-seq and scSeq data. It could also predict immune infiltration and T cell-glioma cell interaction via IFNGR1 pathway. These advantages make it a tool better than classic pathological grading in aspect of studying antitumor immunity.

LncRNAs are commonly considered as transcripts that cannot code peptides, but some small peptides encoded by lncRNAs were recently discovered, making it a new and interesting field in the study of non-coding RNAs (ncRNAs). Some of the m6A modification sites were found to participate in regulating ncRNA translation as elements similar to internal ribosome entry sites [21, 54]. Moreover, these small peptides are also involved in the progression of different kinds of cancers. For example, the micropeptide 53aa encoded by the lncRNA HOXB-AS3 plays a role in metabolic reprogramming in colon cancer, and it suppresses the proliferation of colon cancer cells [55]. Another study showed that a 130aa-peptide known as SRSP encoded by the lncRNA LOC90024 plays a role in modulating mRNA splicing by binding to SRSF3 and thereby promoting the proliferation, migration, and invasion of tumor cells [56]. The current study has also proven that Yin Yang 1-binding micropeptide (YY1BM) encoded by LINC00278 can be more efficiently translated when m6A modification is demethylated by ALKBH5, and it protects esophageal squamous carcinoma cells from apoptosis induced by nutrient deprivation [24]. Thus, peptides translated from lncRNA have a strong effect on cancer cell biology and can be regulated by RNA modification such as m6A. In our study, we found non-m6A modification model exhibited better prognostic efficacy compared with m6A modification model according to TransLnc database. In addition, lncRNA-encoded micropeptides have been proven to affect immunity functions. The lncRNA Aw112010 encodes a small peptide in murine macrophages, which promotes inflammation in mucosa immunity [57]. Another peptide, miPEP155 encoded by MIR155HG, has been reported to affect antigen presentation by binding to a chaperone: HSC70 [58, 59]. Furthermore, the antigen encoded by lncRNAs can also be present and involved in cellular immunosurveillance [17]. Moreover, the m6A reader YTHDF1 is involved in promoting neoantigen degradation by regulating levels of lysosomal proteases through translation [60]. This is consistent with our results in that NAS is positively associated with immune infiltration and T-cell-induced immunity as well as with antigen presentation pathways. The TransLnc database [25] collected all predicted encoded peptides from lncRNAs together with the possible m6A modification status of lncRNAs. The binding affinities of these peptides to MHC complexes were also calculated using NetMHCpan [61], which suggests that RNA modification plays a significant role in antigen production from lncRNA. Our work confirms that the signature of several neoantigen-coding lncRNAs from TransLnc correlates with glioma patients’ survival outcomes. Additionally, the neoantigen peptides provided by TransLnc were predicted to have a high probability of binding to potential neoantigen-reactive TCR clonotypes, which might be potential therapeutic targets for immunotherapy.

For tumors with immunosuppressive microenvironments such as gliomas [62], canonical immunotherapies have not exhibited satisfying outcomes in primary gliomas, and some such therapies have only benefited patients with recurrent gliomas [3–7]. Our study shows that tumor-related inflammation was inhibited in high-grade and high NAS glioma groups even though immune infiltration, including the level of CD8 + T cells, increased. IHC results also revealed higher PD-L1 levels in higher NAS gliomas. Therefore, innovations in gliomas immunotherapies are urgently required. The neoantigens belong to immunopeptides that specifically present on the surface of tumor cells. Due to its specificity in tumor immunity, these are considered promising targets for immunotherapy [63]. Recently, the neoantigen vaccine’s ability to induce T-cell-mediated immunity has been widely discussed [9, 63, 64]. Some circumstantial evidence advocates for the reinvigoration of exhausted T cells in tumor patients. An in vivo experiment showed that the vaccination can achieve beneficial outcomes when neoantigen-reactive T cells express exhausted markers before vaccination [65]. Despite the fact that it was difficult to reinvigorate exhausted T cells after vaccination, it was suggested that, even after a tumor grows and possible immunosuppression is established, T-cell-mediated anti-tumor immunity can be facilitated by neoantigen vaccination. Our results suggest that non-m6A-related neoantigen-coding lncRNAs play a crucial role in determining glioma prognosis, and the study screens widely existing unique TCR clonotypes that could recognize potential neoantigens encoded by selected lncRNAs. High NAS gliomas are also found to contain more neoantigen-reactive TCR patterns, indicating NAS model together with lncRNA-derived micropeptides is associated with neoantigen-related immune response. The identified TCR-neoantigen pairs could provide universal targets for CAR-T therapy. Moreover, the protein-coding abilities of selected lncRNAs and the binding ability of potential micropeptides to TCR might be validated in further researches.

Our study established a NAS model based on non-m6A-related lncRNAs that were predicted to encode neoantigen peptides. This model exhibited its abilities to predict prognosis of glioma patients, immune infiltration in gliomas and neoantigen expression in tumor vaccine therapy. The correlations between NAS-related genes and PD-L1 was also verified by IHC. Besides, the screening of TCR-neoantigen binding pairs also provided several neoantigen-reactive TCR patterns that might be utilized for CAR-T therapy. But the effect of these TCR patterns needs more biological experiment verification in the future.

## Conclusions

In conclusion, we established a prognostic model based on the non-m6A-related neoantigen-coding lncRNAs and NAS, which were found to be positively correlated with T cell immunity, antigen processing and presentation, and immune infiltration. We also screened possible TCR clonotypes of universally targeted neoantigens that were translated from the selected lncRNAs. Therefore, this study details the important role of non-m6A modification in peptides encoded by lncRNA, and it provides TCR clonotypes that can be used in potential CAR-T studies and therapies in the future.

## Supplementary Information

**Additional file 1: Fig. S1.** Selection of non-m6A-related neoantigen-coding lncRNAs in TCGA dataset. **A** The overall workflow of this study. **B** The correlation network between non-m6A regulators and related lncRNAs. **C** The p value of survival analysis and hazard ratio of 13 prognosis-related neoantigen coding lncRNAs. **D** Detailed expression of 13 prognosis-related neoantigen coding lncRNAs in normal and glioma samples illustrated by boxplot. **E** Comparison of non-m6A-related NAS models and other prognostic models with ROC curves in all three RNA-seq datasets (TCGA, CGGA325, CGGA693).

**Additional file 2: Fig. S2.** Construction procedures of cluster model with non-m6A-related lncRNAs. **A** Left, consensus matrices of the TCGA samples, showing consensus matrices with k =2-5. Right, the cluster-consensus value for k = 2-9. The subcolumns indicate cluster-consensus values of different clusters under different k value. **B** PCA plot showing the distribution of cluter1 and cluster2 samples. **C, D **Expression of non-m6A regulators in CGGA325 **(C) **and CGA693 **(D) **datasets illustrated by heatmaps based on clinical features and NAS. **E** Venn diagram showing the distribution of DEGs with significant prognostic effect.

**Additional file 3: Fig. S3.** The survival analyses of TCGA, CGGA325 and CGGA693 in all gliomas, GBM and LGG, respectively, based on cluster model.

**Additional file 4: Fig. S4.** The survival analyses of TCGA, CGGA325 and CGGA693 in all gliomas and LGG, respectively, based on NAS.

**Additional file 5: Fig. S5.** Construction of cluster model based on m6A-related lncRNAs. **A** Left, consensus matrices of the TCGA samples, showing consensus matrices with k =2-5. Right, the cluster-consensus value for k = 2-9. The subcolumns indicate cluster-consensus values of different clusters under different k value. **B** PCA plot showing the distribution of cluter1 and cluster2 samples. **C** The p value of survival analysis and hazard ratio of 13 prognosis-related neoantigen coding lncRNAs. **D** Detailed expression of 13 prognosis-related neoantigen coding lncRNAs in normal and glioma samples illustrated by boxplot.

**Additional file 6: Fig. S6.** The details in different cell clusters in scSeq datasets. **A, B** The expression of marker genes for annotation in different clusters in GSE84465 **(A)** and CGGA **(B)** dataset. **C, D** The expression of highly characteristic marker genes in different clusters in GSE84465 **(C)** and CGGA **(D)** dataset. **E, F** The pseudotime analyses of GSE84465 **(E)** and CGGA **(F)** datasets. Higher pseudotime index suggested a more downstream location of cells. **G, H** The cell trajectory analyses of different cell clusters in GSE84465 **(G)** and CGGA **(H)** dataset. **I** The latent time analysis of RNA velocity in GSE84465.

**Additional file 7: Fig. S7.** The details of immune landscape in CGGA325 and CGGA693 datasets. **A, B** Correlations between Stromal score, ESTIMATE score, Immune score, tumor purity and NAS in CGGA325 **(A)** and CGGA693 **(B)**. **C, D** Infiltration ratio of all immunocytes in low and high NAS groups of CGGA325 analyzed by CIBERSORT **(C)**. Infiltration ratio of Treg, CD8+ T cells, T helper and activated NK cells were manifested **(D)**. **E, F** Infiltration ratio of all immunocytes in low and high NAS groups of CGGA693 analyzed by CIBERSORT **(E)**. Infiltration ratio of Treg, CD8+ T cells, T helper and activated NK cells were manifested **(F)**. **G** The genes with top 10 PC1+PC2 values in TCGA dataset.

**Additional file 8: Fig. S8.** The details of expression of positive regulators of T cells in CGGA325 and CGGA693 and SNV data of positive regulators of T cells in TCGA clustering model. **A, B** The expression of positive regulators of T cells in CGGA325 **(A)** and CGGA693 **(B)** dataset illustrated by heatmaps based on clinical features and NAS. **C, D** Single nucleotide variations of positive regulators of T cells in cluster 1 **(C)** or cluster 2 **(D)** in TCGA dataset, respectively. **E** Workflow of the Jurkar-LN229 co-culture assay.

**Additional file 9: Fig. S9.** The top 5 activated transcription factors analyzed by pySCENIC in low and high NAS groups in CGGA scSeq dataset **(A)** and GSE129671 **(B)**.

**Additional file 10: Fig. S10**. Intercellular communication analysis of single cell RNA-seq based on CGGA scSeq dataset. **A, B** Top 30 interactions between cells in low **(A)** or high **(B)** NAS groups, respectively. **C** The interaction ratios between T cells and four kinds of glioma cells in single cell RNA-seq of low or high neoantigen score groups. **D-F** The expression of IFNGR1 **(D)**, JAK1 **(E)** and JAK2 **(F)** in four kinds of glioma cells. **G** The correlations between expression of IFNGR1, JAK1, JAK2 and neoantigen activation scores in glioma cells.

**Additional file 11: Fig. S11.** The details in TCR clonotype analysis of GSE188620 (scTCR data). **A** The amount and percent of unique TCR clonotypes in four patients before and after vaccination. **B** The number of unique TCR clonotypes with different abundances in four patients before and after vaccination. **C** The length distribution of TCR clonotypes displayed by the numbers of CDR3 amino acids in different kinds of CDR3 length. Total clonotypes or clonotypes in α, β chains are shown. **D** The clonal diversity of TCR clonotypes in total or every sample before and after vaccination. **E** The NAS in RNA-seq data of unvaccinated or vaccinated gliomas.

**Additional file 12: Table S1.** DEGs identified for non-m6A-related NAS model construction.

**Additional file 13: Table S2.** DEGs identified for m6A-related NAS model construction.

**Additional file 14: Table S3.** Primer sequences used for qRT-PCR.

**Additional file 15: Table S4.** Identified regulators in Ψ, m5C and m1A.

**Additional file 16: Table S5.** The correlations between non-m6A or m6A regulators and related-lncRNAs.

**Additional file 17: Table S6.** DEGs identified for non-m6A-related NAS model construction in scSeq data.

**Additional file 18: Table S7.** Chi-square test of mutation rate of T-cell positive regulators in low/high NAS groups and cluter1/2.

**Additional file 19: Table S8.** Identified common TCR patterns in GBM and LGG samples.

## Data Availability

The data utilized in study were downloaded from TCGA Research Network portal (https://portal.gdc.cancer.gov/), Gene Expression Omnibus (GEO, http://www.ncbi.nlm.nih.gov/geo/) and Chinese Glioma Genome Atlas (CGGA, http://www.cgga.org.cn/). The relative codes could by supplied upon reasonable request.

## Abbreviations

LncRNAs: Long non-coding RNAs
NAS: Neoantigen activation score
TCR: T cell receptor
CAR-T: Chimeric antigen receptor T cell
PD-1: Programmed death-1
PD-L1: Programmed death-ligand 1
IFNGR1: Interferon gamma receptor 1
JAK1: Janus kinase 1
JAK2: Janus kinase 2
SNV: Single nucleotide variations
smORF or sORF: Small or short open-reading frames
eIF4E: Eukaryotic translation initiation factor 4E
m6A: N6-methyladenosine
Ψ: Pseudouridine
m1A: N1-methyladenosine
m5C: 5-Methylcytosine
MHC I: Major histocompatibility complex class 1 molecules
MHC II: Major histocompatibility complex class 2 molecules
TMB: Tumor mutation burden
GBM: Glioblastoma multiforme
LGG: Low-grade glioma
TCGA: The Cancer Genome Atlas
CGGA: Chinese Glioma Genome Atlas
scSeq: Single-cell sequencing
GEO: Gene Expression Omnibus
WHO: World Health Organization
IHC: Immunohistochemistry
TMSB10: Thymosin beta 10
VIM: Vimentin
DAB: 3, 3′-Diaminobenzidine
DEG: Differential expression gene
SVM: Support vector machine
FPKM: Kilobase of transcript per million mapped reads
HR: Hazard ratio
ROC: Receiver operation characteristic
AUC: Area under the curve
GSVA: Gene set variation analysis
GO: Gene ontology
KEGG: Kyoto Encyclopedia of Genes and Genomes
GSEA: Gene set enrichment analysis
RNA-seq: RNA sequencing
X2K: Expression2Kinases
ANOVA: Analysis of variance
ac4C: N4-acetylcytidine
Nm: 2'-O-methylation
m7G: 7-Methylguanosine
FTO: Fat mass and obesity-associated gene
TET2: Tet methylcytosine dioxygenase 2
ALKBH1: AlkB homolog 1
ALKBH3: AlkB homolog 3
NMF: Non-negative matrix factorization
MES: Mesenchymal
PN: Proneural
t-SNE: T-distributed stochastic neighbor embedding
OPC: Oligodendrocyte-progenitor-like
NK: Natural killer
AHNAK: AHNAK Nucleoprotein
CALML3: Calmodulin-like 3
ZNF830: Zinc finger protein 830
HOMER1: Homer protein homolog 1
NFYB: Nuclear transcription factor Y subunit beta
AKR1C4: Aldo–keto reductase
SUZ12: Polycomb repressive complex 2 subunit
EZH2: Enhancer of zeste homolog 2
SMAD4: SMAD family member 4
AR: Androgen receptor
SOX8: SRY-box transcription factor 8
SOX10: SRY-box transcription factor 10
EGR1: Early growth response 1
ADAM12: ADAM metallopeptidase domain 12
ITGA9: Integrin, alpha 9
SDC4: Syndecan 4
CDR3: Complementarity determining region 3
ncRNAs: Non-coding RNAs
YY1BM: Yin Yang 1-binding micropeptide
HOXB-AS3: HOXB cluster antisense RNA 3
SRSP: Splicing regulatory small protein
SRSF3: Serine- and arginine-rich splicing factor 3
ALKBH5: AlkB homolog 5
HSC70: Heat shock cognate protein 70
YTHDF1: YTH N6-methyladenosine RNA binding protein 1
