# Supplementary figures and images for: Scoring model based on the signature of non-m6A-related neoantigen-coding lncRNAs assists in immune microenvironment analysis and TCR-neoantigen pair selection in gliomas
Source: J Transl Med. 2022 Oct 29;20:494. doi: 10.1186/s12967-022-03713-z (PMC9617417; doi:10.1186/s12967-022-03713-z)

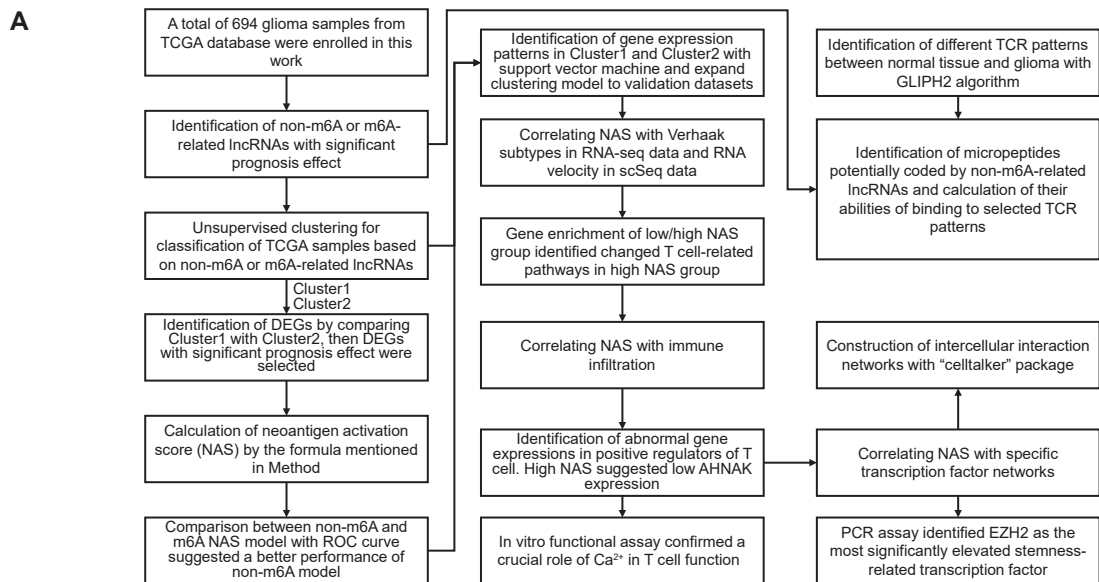

**B**

● lncRNA ● non-m6A regulators

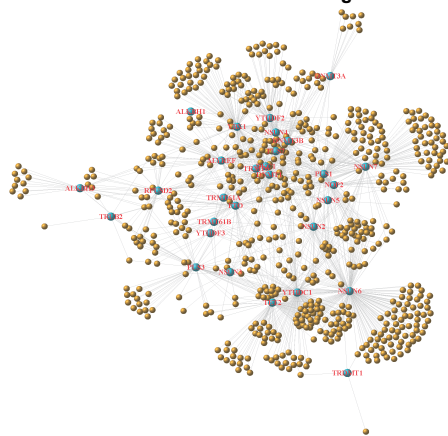

**C**

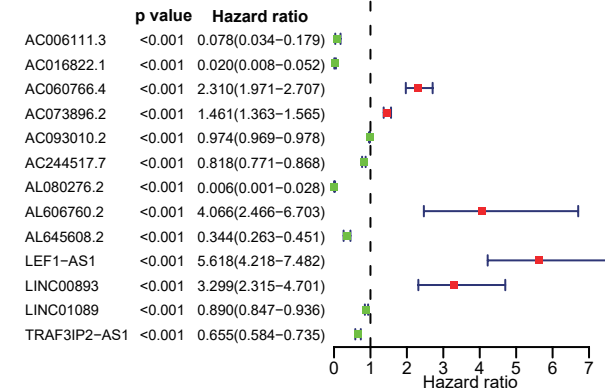

**D**

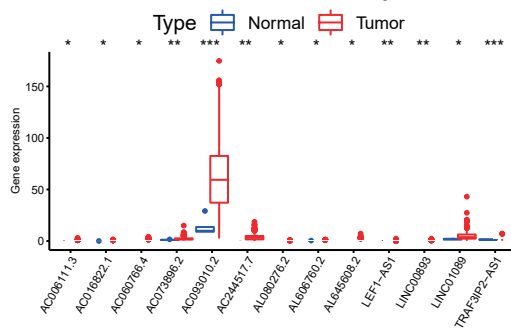

**E**

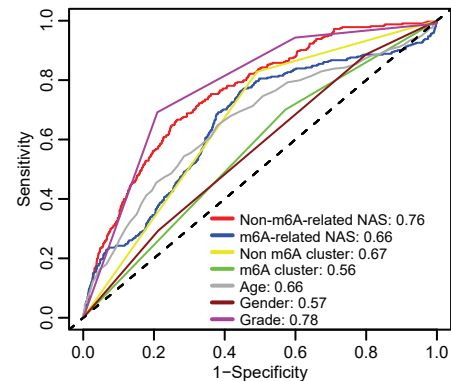

Supplement: Supplementary file 1 — Additional file 1: Fig. S1. Selection of non-m6A-related neoantigen-coding lncRNAs in TCGA dataset. A The overall workflow of this study. B The correlation network between non-m6A regulators and related lncRNAs. C The p value of survival analysis and hazard ratio of 13 prognosis-related neoantigen coding lncRNAs. D Detailed expression of 13 prognosis-related neoantigen coding lncRNAs in normal and glioma samples illustrated by boxplot. E Comparison of non-m6A-related NAS models and other prognostic models with ROC curves in all three RNA-seq datasets (TCGA, CGGA325, CGGA693). [file 12967_2022_3713_MOESM1_ESM.pdf]

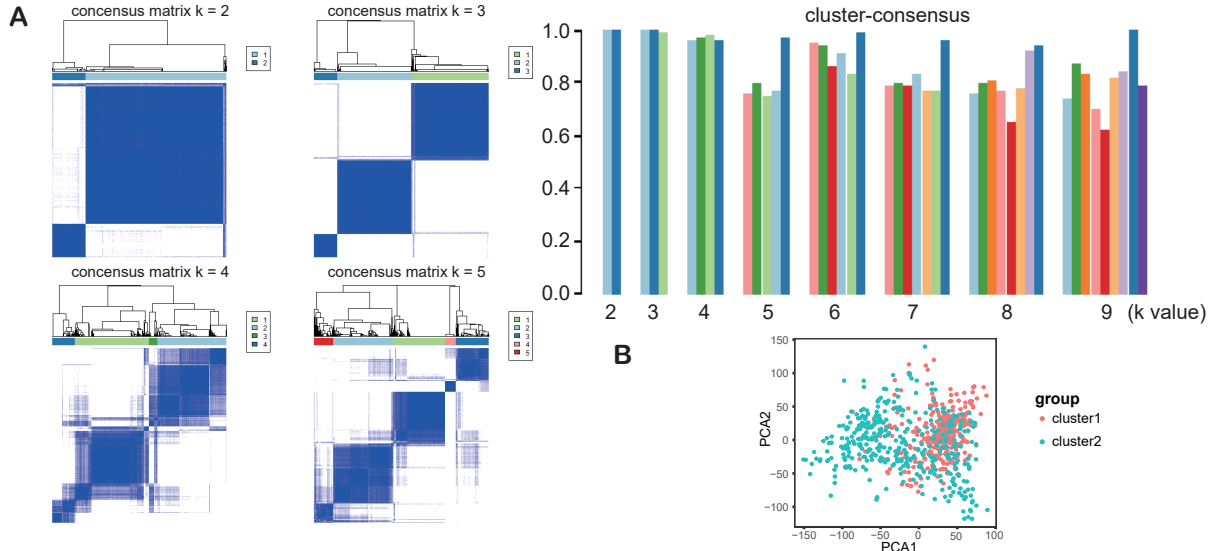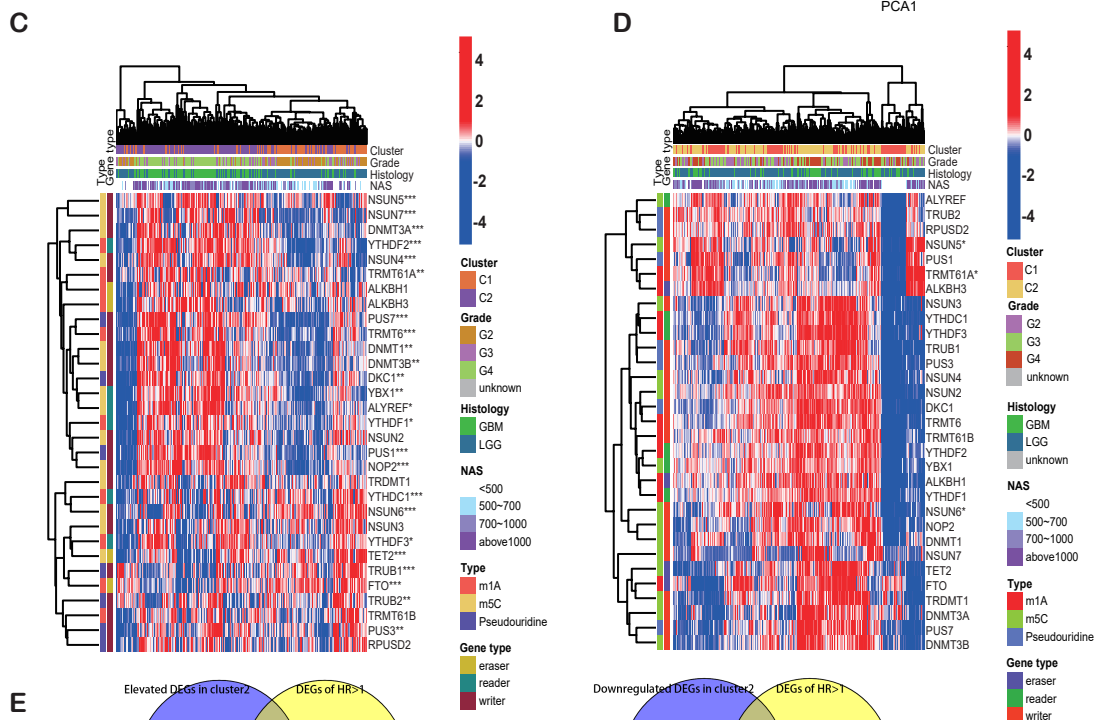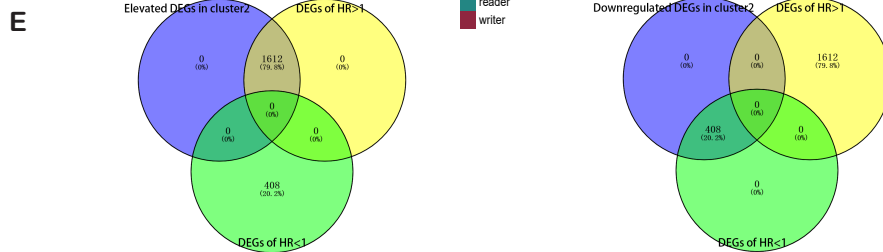

Supplement: Supplementary file 2 — Additional file 2: Fig. S2. Construction procedures of cluster model with non-m6A-related lncRNAs. A Left, consensus matrices of the TCGA samples, showing consensus matrices with k =2-5. Right, the cluster-consensus value for k = 2-9. The subcolumns indicate cluster-consensus values of different clusters under different k value. B PCA plot showing the distribution of cluter1 and cluster2 samples. C, D Expression of non-m6A regulators in CGGA325 (C) and CGA693 (D) datasets illustrated by heatmaps based on clinical features and NAS. E Venn diagram showing the distribution of DEGs with significant prognostic effect. [file 12967_2022_3713_MOESM2_ESM.pdf]

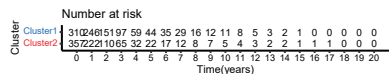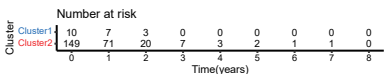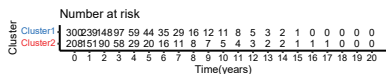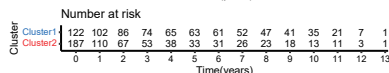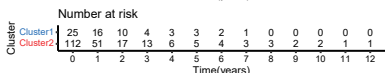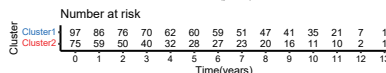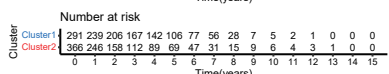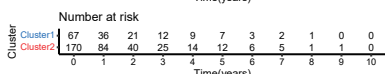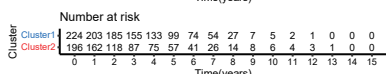

Supplement: Supplementary file 3 — Additional file 3: Fig. S3. The survival analyses of TCGA, CGGA325 and CGGA693 in all gliomas, GBM and LGG, respectively, based on cluster model. [file 12967_2022_3713_MOESM3_ESM.pdf]

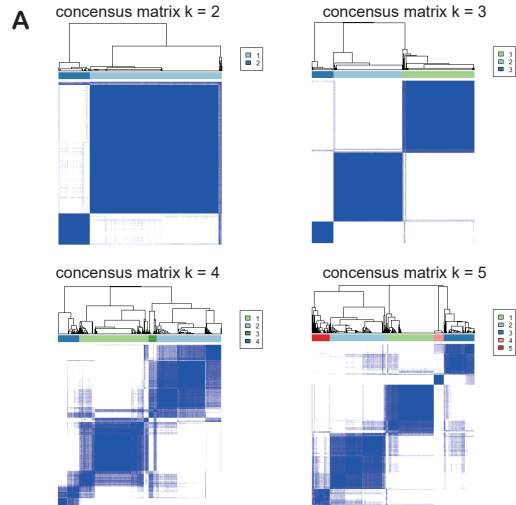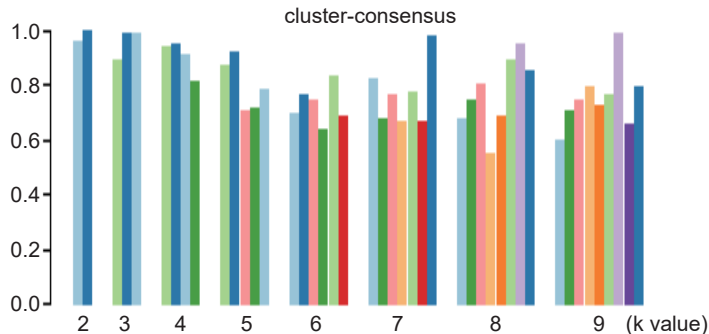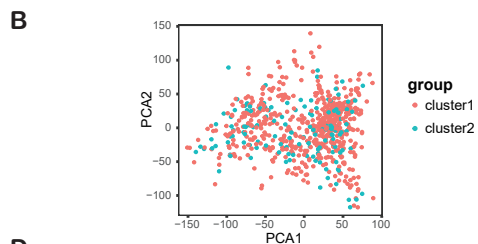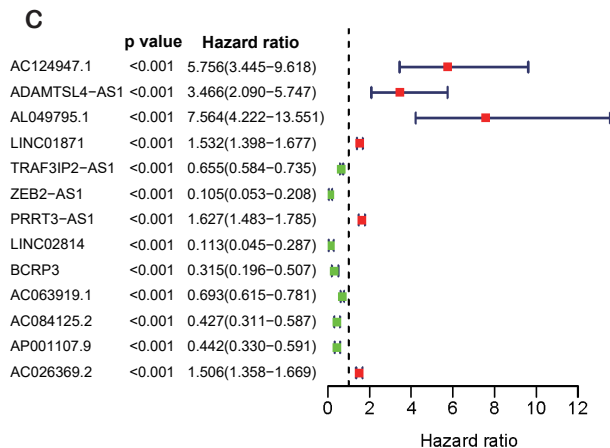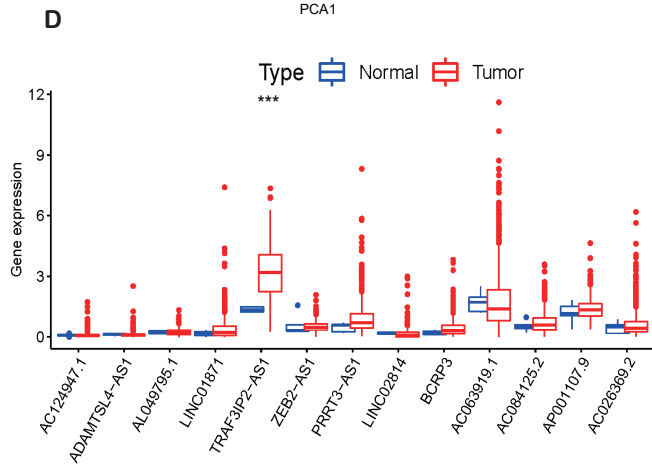

Supplement: Supplementary file 5 — Additional file 5: Fig. S5. Construction of cluster model based on m6A-related lncRNAs. A Left, consensus matrices of the TCGA samples, showing consensus matrices with k =2-5. Right, the cluster-consensus value for k = 2-9. The subcolumns indicate cluster-consensus values of different clusters under different k value. B PCA plot showing the distribution of cluter1 and cluster2 samples. C The p value of survival analysis and hazard ratio of 13 prognosis-related neoantigen coding lncRNAs. D Detailed expression of 13 prognosis-related neoantigen coding lncRNAs in normal and glioma samples illustrated by boxplot. [file 12967_2022_3713_MOESM5_ESM.pdf]

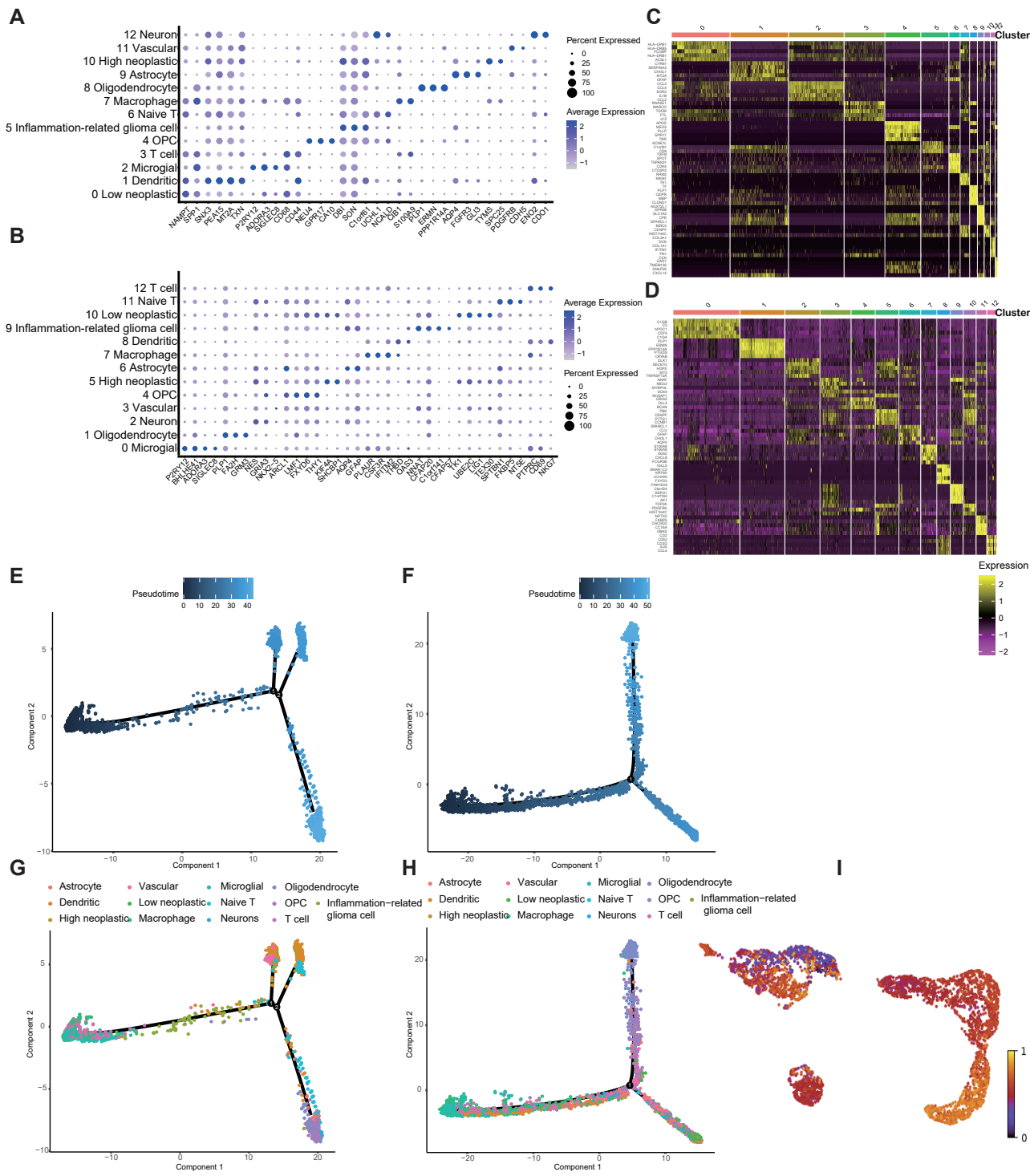

Supplement: Supplementary file 6 — Additional file 6: Fig. S6. The details in different cell clusters in scSeq datasets. A, B The expression of marker genes for annotation in different clusters in GSE84465 (A) and CGGA (B) dataset. C, D The expression of highly characteristic marker genes in different clusters in GSE84465 (C) and CGGA (D) dataset. E, F The pseudotime analyses of GSE84465 (E) and CGGA (F) datasets. Higher pseudotime index suggested a more downstream location of cells. G, H The cell trajectory analyses of different cell clusters in GSE84465 (G) and CGGA (H) dataset. I The latent time analysis of RNA velocity in GSE84465. [file 12967_2022_3713_MOESM6_ESM.pdf]

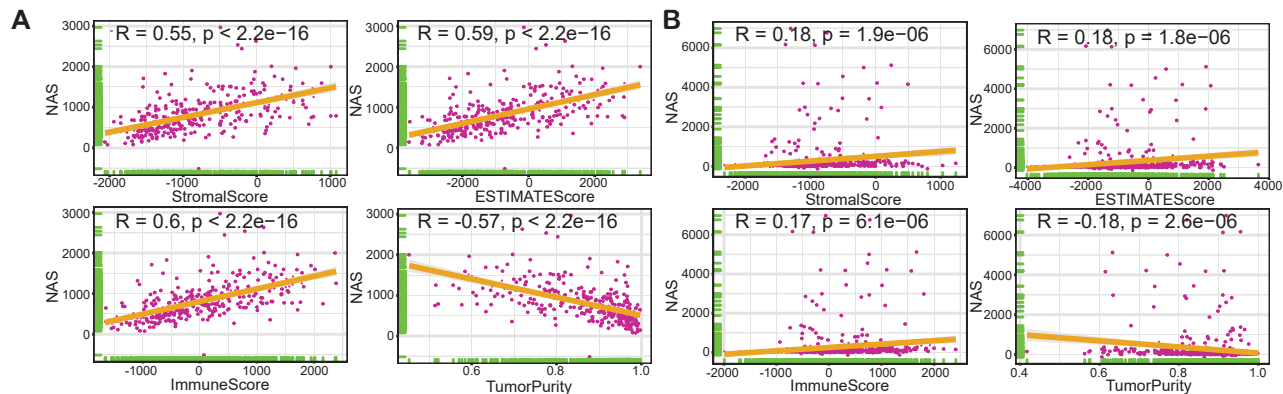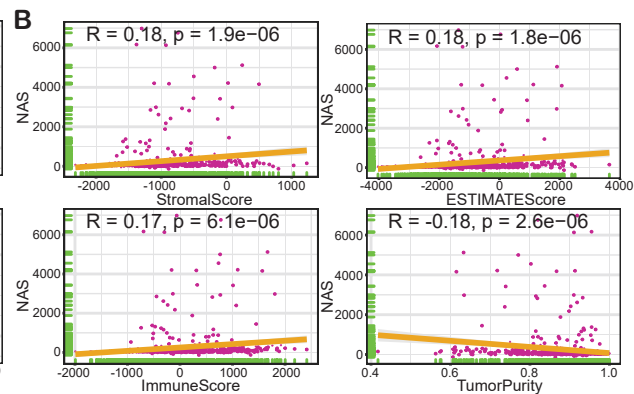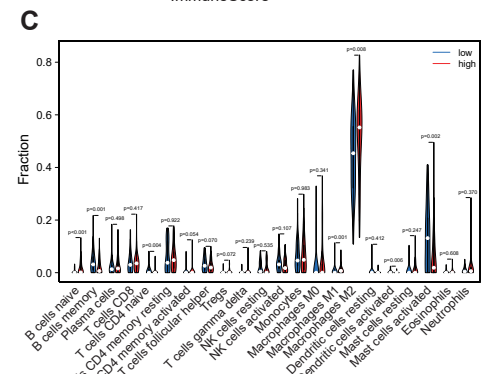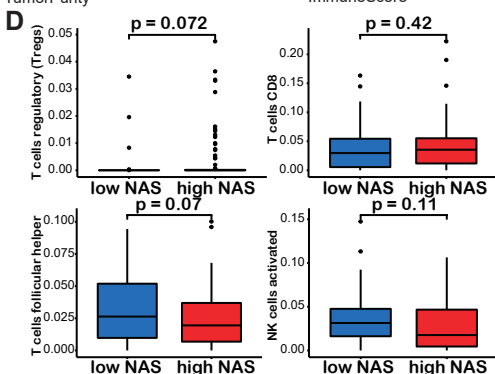

**G**

| Gene          | PC1+PC2     |
|---------------|-------------|
| <b>TMSB10</b> | 603.349147  |
| TMSB4X        | 454.7368611 |
| SPP1          | 382.4859536 |
| CD74          | 257.6634677 |
| HLA-DRA       | 230.243993  |
| CHI3L1        | 215.1306712 |
| <b>VIM</b>    | 202.9118472 |
| AQP1          | 150.8042719 |
| LGALS1        | 103.6522944 |
| HLA-DRB1      | 100.9731173 |

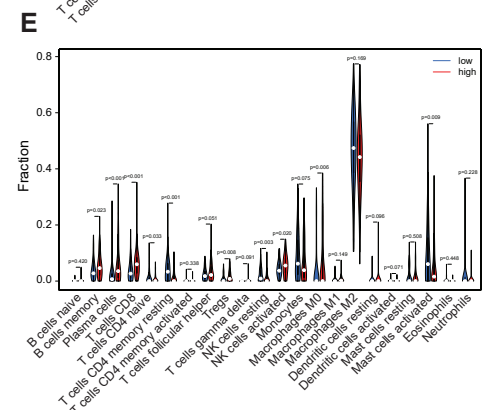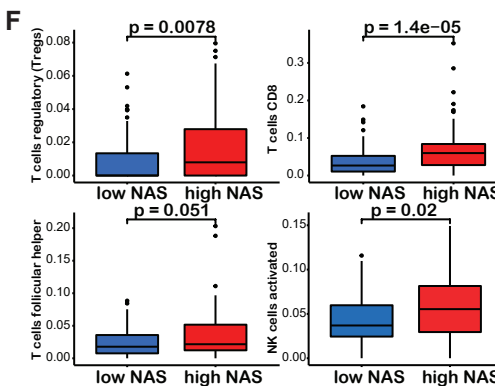

Supplement: Supplementary file 7 — Additional file 7: Fig. S7. The details of immune landscape in CGGA325 and CGGA693 datasets. A, B Correlations between Stromal score, ESTIMATE score, Immune score, tumor purity and NAS in CGGA325 (A) and CGGA693 (B). C, D Infiltration ratio of all immunocytes in low and high NAS groups of CGGA325 analyzed by CIBERSORT (C). Infiltration ratio of Treg, CD8+ T cells, T helper and activated NK cells were manifested (D). E, F Infiltration ratio of all immunocytes in low and high NAS groups of CGGA693 analyzed by CIBERSORT (E). Infiltration ratio of Treg, CD8+ T cells, T helper and activated NK cells were manifested (F). G The genes with top 10 PC1+PC2 values in TCGA dataset. [file 12967_2022_3713_MOESM7_ESM.pdf]

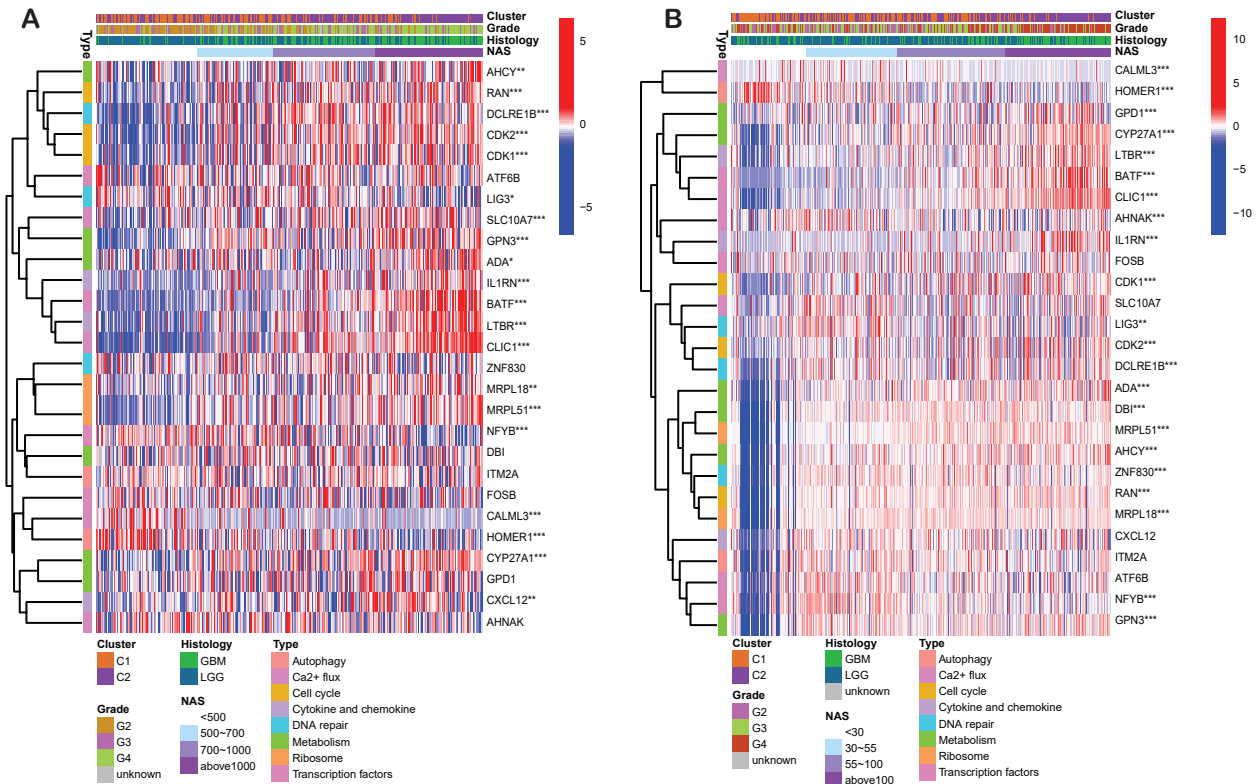

**C** Altered in 19 (6.17%) of 308 samples.

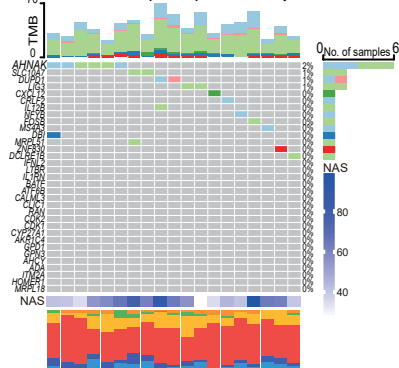

**D** Altered in 29 (8.53%) of 340 samples.

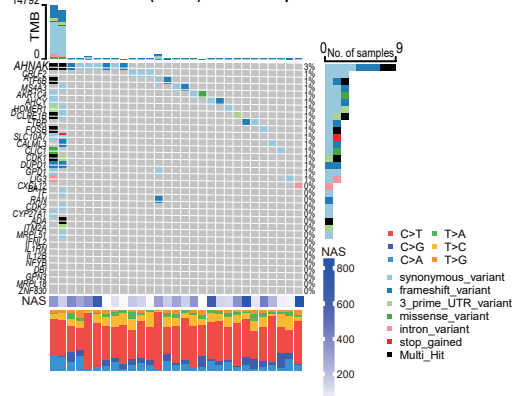

**E**

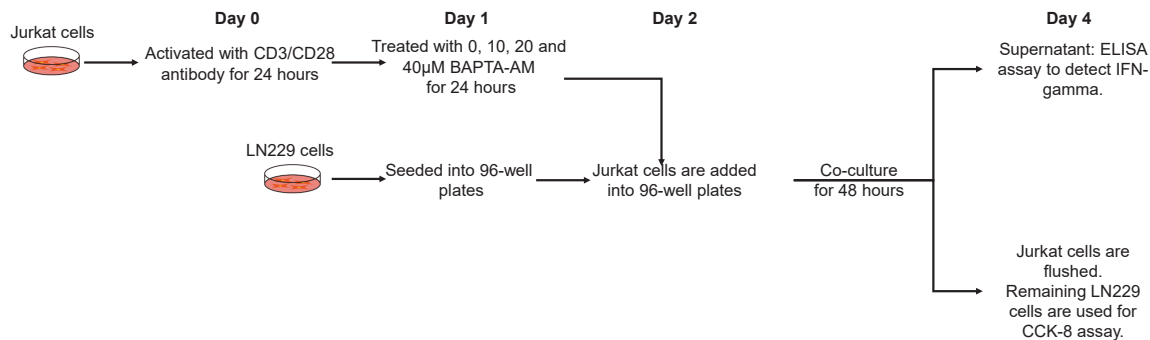

Supplement: Supplementary file 8 — Additional file 8: Fig. S8. The details of expression of positive regulators of T cells in CGGA325 and CGGA693 and SNV data of positive regulators of T cells in TCGA clustering model. A, B The expression of positive regulators of T cells in CGGA325 (A) and CGGA693 (B) dataset illustrated by heatmaps based on clinical features and NAS. C, D Single nucleotide variations of positive regulators of T cells in cluster 1 (C) or cluster 2 (D) in TCGA dataset, respectively. E Workflow of the Jurkar-LN229 co-culture assay. [file 12967_2022_3713_MOESM8_ESM.pdf]

**A**

low NAS

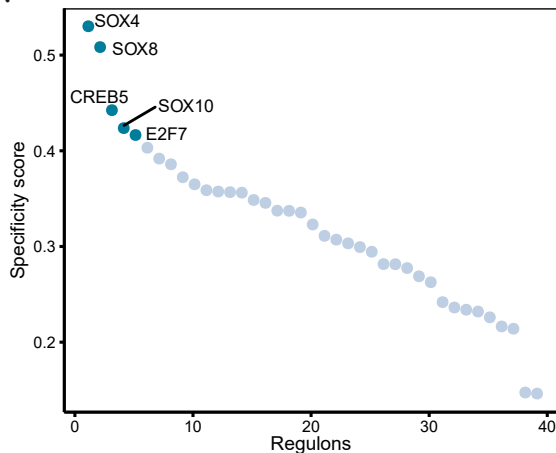

high NAS

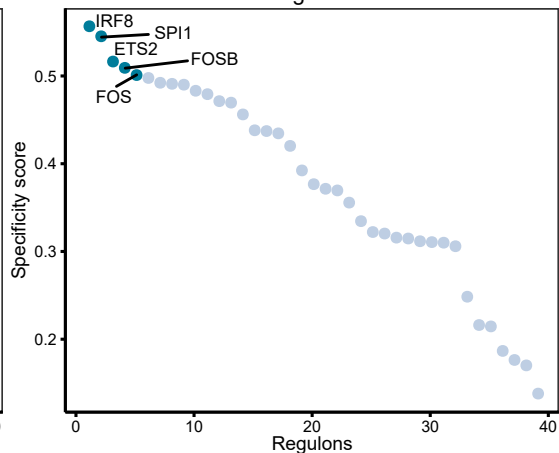**B**

low NAS

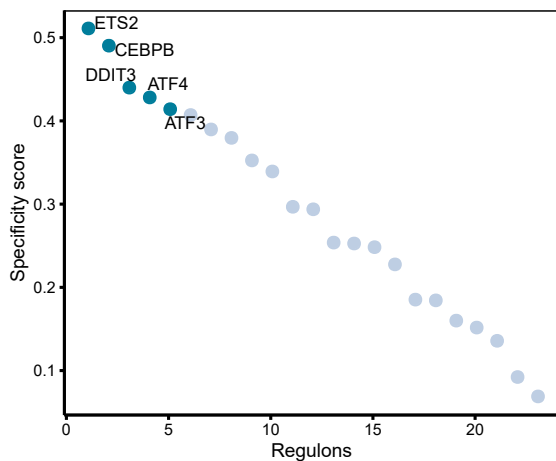

high NAS

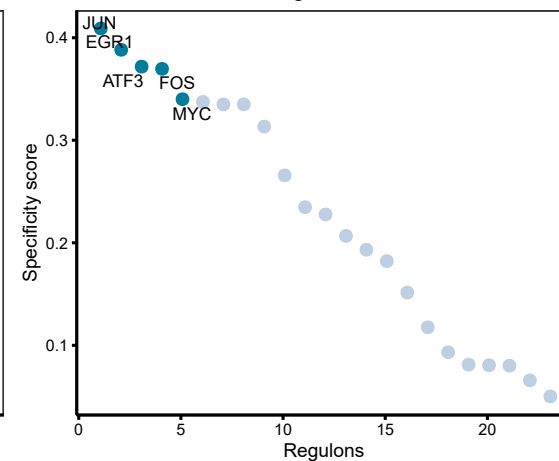

Supplement: Supplementary file 9 — Additional file 9: Fig. S9. The top 5 activated transcription factors analyzed by pySCENIC in low and high NAS groups in CGGA scSeq dataset (A) and GSE129671 (B). [file 12967_2022_3713_MOESM9_ESM.pdf]

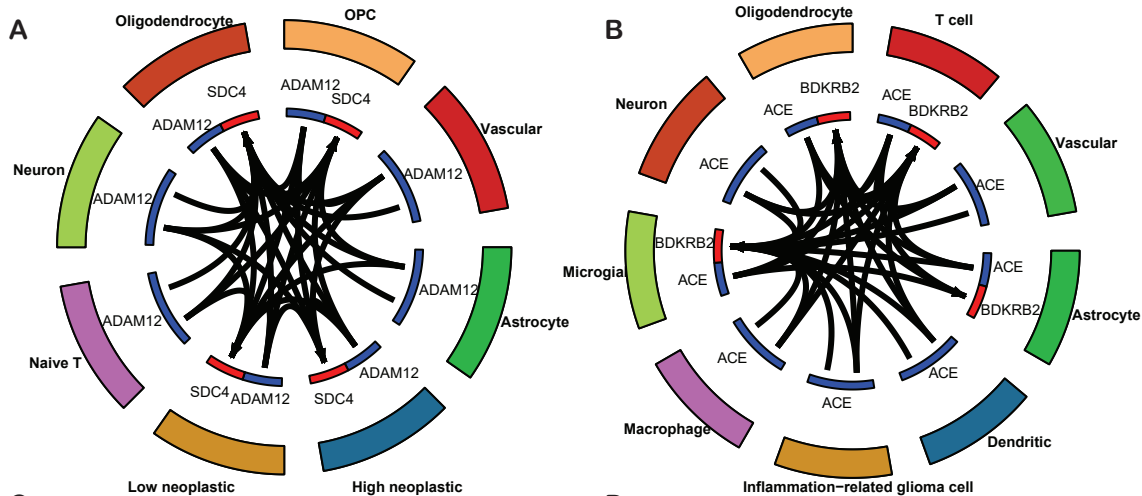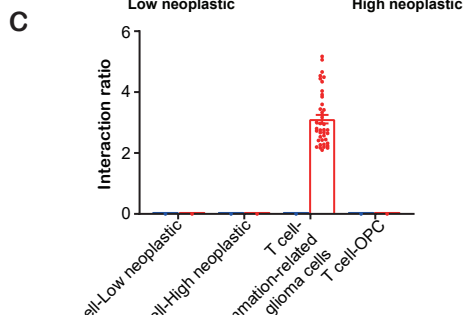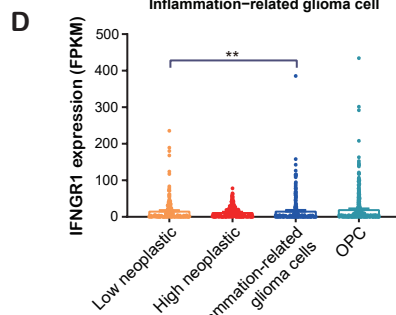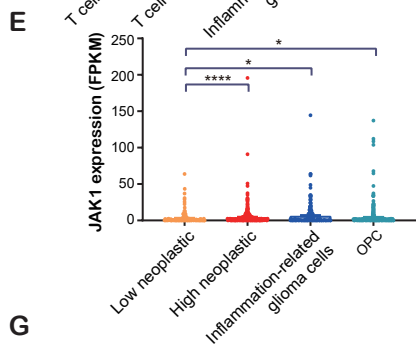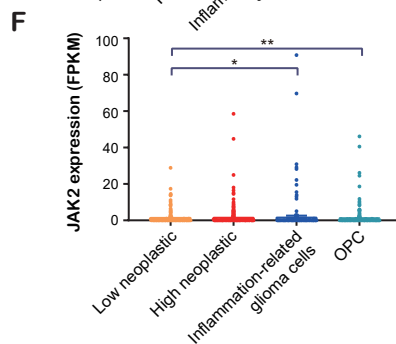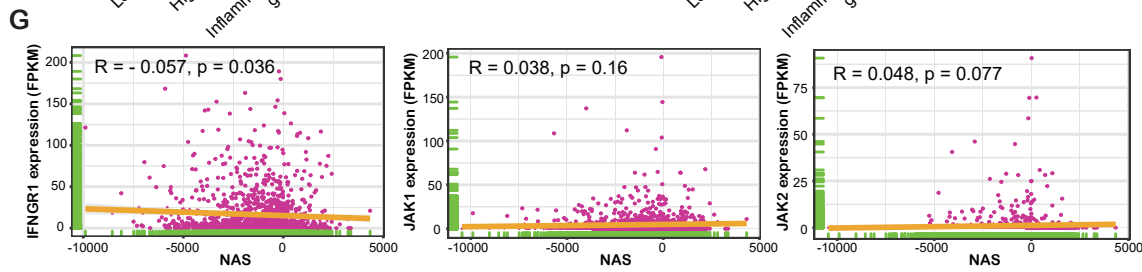

Supplement: Supplementary file 10 — Additional file 10: Fig. S10. Intercellular communication analysis of single cell RNA-seq based on CGGA scSeq dataset. A, B Top 30 interactions between cells in low (A) or high (B) NAS groups, respectively. C The interaction ratios between T cells and four kinds of glioma cells in single cell RNA-seq of low or high neoantigen score groups. D-F The expression of IFNGR1 (D), JAK1 (E) and JAK2 (F) in four kinds of glioma cells. G The correlations between expression of IFNGR1, JAK1, JAK2 and neoantigen activation scores in glioma cells. [file 12967_2022_3713_MOESM10_ESM.pdf]

**A**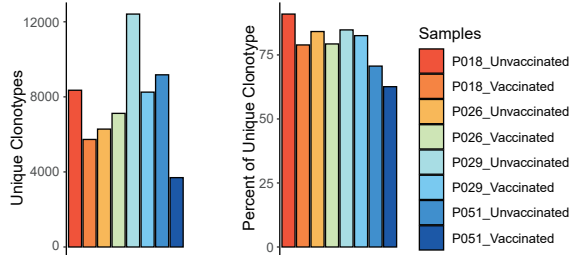**B**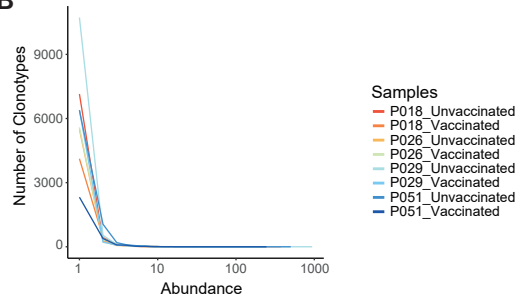**C**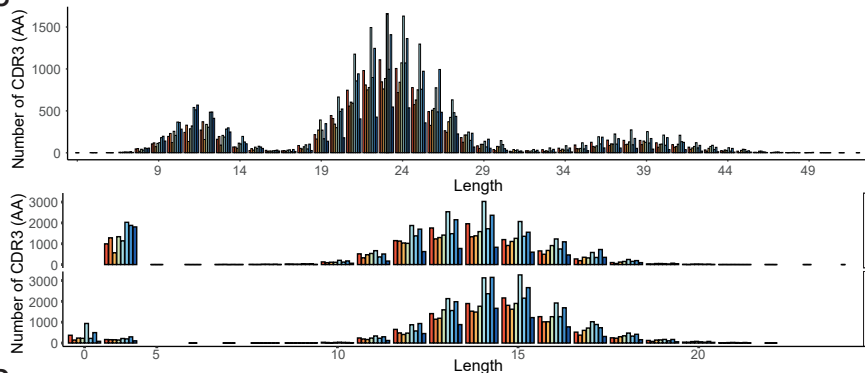**D**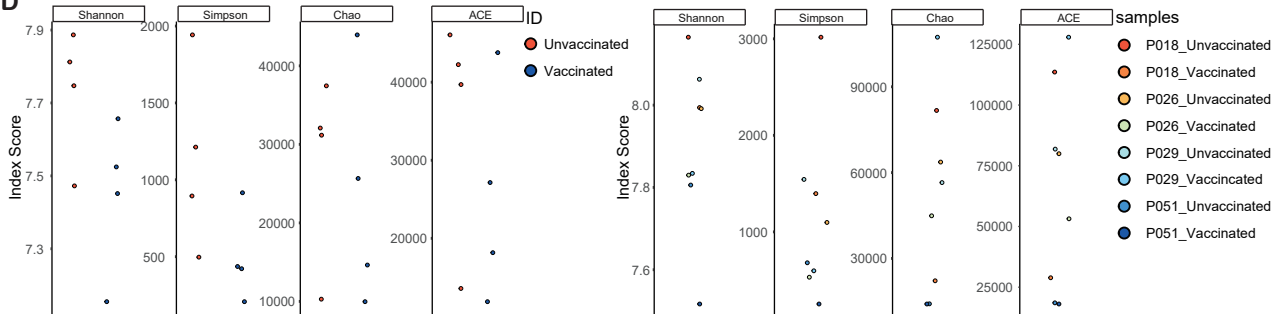

Supplement: Supplementary file 11 — Additional file 11: Fig. S11. The details in TCR clonotype analysis of GSE188620 (scTCR data). A The amount and percent of unique TCR clonotypes in four patients before and after vaccination. B The number of unique TCR clonotypes with different abundances in four patients before and after vaccination. C The length distribution of TCR clonotypes displayed by the numbers of CDR3 amino acids in different kinds of CDR3 length. Total clonotypes or clonotypes in α, β chains are shown. D The clonal diversity of TCR clonotypes in total or every sample before and after vaccination. E The NAS in RNA-seq data of unvaccinated or vaccinated gliomas. [file 12967_2022_3713_MOESM11_ESM.pdf]
