# Supplementary material for: Scoring model based on the signature of non-m6A-related neoantigen-coding lncRNAs assists in immune microenvironment analysis and TCR-neoantigen pair selection in gliomas
Source: J Transl Med. 2022 Oct 29;20:494. doi: 10.1186/s12967-022-03713-z (PMC9617417; doi:10.1186/s12967-022-03713-z)
Supplement: Supplementary file 4 — Additional file 4: Fig. S4. The survival analyses of TCGA, CGGA325 and CGGA693 in all gliomas and LGG, respectively, based on NAS. [file 12967_2022_3713_MOESM4_ESM.pdf]

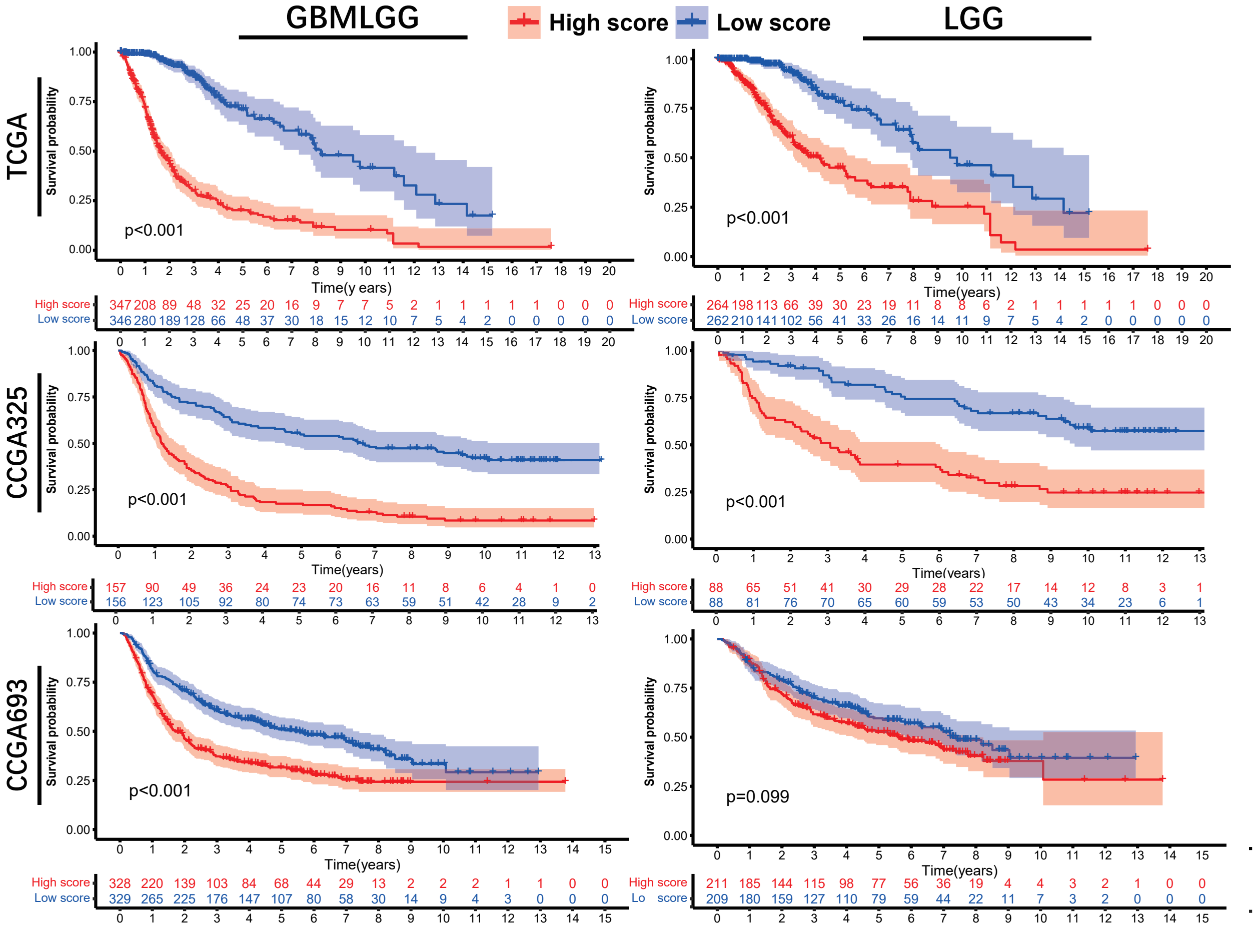

GBMLGG
High score
Low score
LGG
TCGA
CCGA325
CCGA693
Survival probability
1.00
0.75
0.50
0.25
0.00
p<0.001
p<0.001
p<0.001
p<0.001
p<0.001
p=0.099
Time(y ears)
Time(years)
High score 347 208 89 48 32 25 20 16 9 7 7 5 2 1 1 1 1 1 0 0 0
Low score 346 280 189 128 66 48 37 30 18 15 12 10 7 5 4 2 0 0 0 0 0
High score 264 198 113 66 39 30 23 19 11 8 8 6 2 1 1 1 1 1 0 0 0
Low score 262 210 141 102 56 41 33 26 16 14 11 9 7 5 4 2 0 0 0 0 0
High score 157 90 49 36 24 23 20 16 11 8 6 4 1 0
Low score 156 123 105 92 80 74 73 63 59 51 42 28 9 2
High score 88 65 51 41 30 29 28 22 17 14 12 8 3 1
Low score 88 81 76 70 65 60 59 53 50 43 34 23 6 1
High score 328 220 139 103 84 68 44 29 13 2 2 2 1 1 0 0
Low score 329 265 225 176 147 107 80 58 30 14 9 4 3 0 0 0
High score 211 185 144 115 98 77 56 36 19 4 4 3 2 1 0 0
Lo score 209 180 159 127 110 79 59 44 22 11 7 3 2 0 0 0
